# Structural proteins of human coronaviruses: what makes them different?

**DOI:** 10.3389/fcimb.2024.1458383

**Published:** 2024-12-06

**Authors:** Nail Minigulov, Kuandyk Boranbayev, Ayaulym Bekbossynova, Bakhytgul Gadilgereyeva, Olena Filchakova

**Affiliations:** Biology Department, School of Sciences and Humanities, Nazarbayev University, Astana, Kazakhstan

**Keywords:** coronaviruses, structural proteins, SARS, MERS, HKU1, OC43, NL63, 229E

## Abstract

Following COVID-19 outbreak with its unprecedented effect on the entire world, the interest to the coronaviruses increased. The causative agent of the COVID-19, severe acute respiratory syndrome coronavirus – 2 (SARS-CoV-2) is one of seven coronaviruses that is pathogenic to humans. Others include SARS-CoV, MERS-CoV, HCoV-HKU1, HCoV-OC43, HCoV-NL63 and HCoV-229E. The viruses differ in their pathogenicity. SARS-CoV, MERS-CoV, and SARS-CoV-2 are capable to spread rapidly and cause epidemic, while HCoV-HKU1, HCoV-OC43, HCoV-NL63 and HCoV-229E cause mild respiratory disease. The difference in the viral behavior is due to structural and functional differences. All seven human coronaviruses possess four structural proteins: spike, envelope, membrane, and nucleocapsid. Spike protein with its receptor binding domain is crucial for the entry to the host cell, where different receptors on the host cell are recruited by different viruses. Envelope protein plays important role in viral assembly, and following cellular entry, contributes to immune response. Membrane protein is an abundant viral protein, contributing to the assembly and pathogenicity of the virus. Nucleocapsid protein encompasses the viral RNA into ribonucleocapsid, playing important role in viral replication. The present review provides detailed summary of structural and functional characteristics of structural proteins from seven human coronaviruses, and could serve as a practical reference when pathogenic human coronaviruses are compared, and novel treatments are proposed.

## 1 Introduction

Coronaviruses are single-stranded RNA viruses, which belong to the family Coronaviridae. The family consists of four genera: α, β, γ, and δ. There are seven pathogenic to humans coronaviruses: SARS-CoV-2, SARS-CoV, MERS-CoV, HCoV-HKU1, HCoV-OC43, HCoV-NL63, and HCoV-229E. HCoV-NL63 and HCoV-229E belong to α-coronaviruses, the rest are considered β-coronaviruses. HCoV-HKU1, HCoV-OC43, HCoV-NL63, and HCoV-229E usually cause mild or moderate respiratory diseases, while SARS-CoV-2, SARS-CoV, and MERS-CoV cause severe respiratory diseases. The viruses differ in their replication potential, with SARS-CoV-2 virus outperforming other viruses, and having high spreading potential. SARS-CoV caused severe acute respiratory syndrome (SARS) outbreak in 2003 leading to over 8000 cases with fatality rate of 11% (World Health Organization, 2003). The Middle East Respiratory Syndrome (MERS) is a viral respiratory disease caused by the Middle East Respiratory Syndrome Coronavirus (MERS‐CoV). This virus was identified in Saudi Arabia in 2012 (Bermingham et al., 2012). MERS-CoV can cause a variety of symptoms and сlinical manifestations that range from mild to severe, including acute respiratory distress syndrome (ARDS) and organ failure. The latter two conditions are often associated with uncontrolled cytokine production, leading to a cytokine storm. According to WHO, the mortality rate among cases of MERS-CoV disease was 36% (WHO, 2024), which is higher compared to both SARS-CoV and SARS-CoV-2. In comparison, the mortality rate of SARS-CoV-2 and SARS-CoV varies between 2% and 10% (Meo et al., 2020). In contrast, HCoV-NL63, OC43, 229E, HKU1 infections are associated with mild respiratory tract diseases, and there is no extensive data on mortality among those infected. A retrospective cohort study, conducted from October 2012 to December 2017, investigated adults infected with HCoV-229E and HCoV-OC43 coronaviruses, and suggested that HCoV-229E is more virulent compared to HCoV-OC43. The study reported a 30-day all-cause mortality rate of 25% for patients infected with HCoV-229E and 9.1% for HCoV-OC43- infected adult patients (Choi et al., 2021).

There are multiple factors that contribute to the MERS pathogenicity which potentially could explain its higher mortality rate. Among those factors are difference in structural proteins as well as in accessory proteins. For example, MERS is the only coronavirus that utilizes dipeptidyl peptidase IV (DPP4) or CD26 for cellular entry (Wang et al., 2013). Such difference in the target receptor is due to difference in the receptor binding domain of structural protein S (Wang et al., 2013). Within S protein of MERS-CoV there are two furin-cleavage sites which facilitate virus entry into the host cell. Besides this, multiple studies demonstrate that MERS accessory protein ORF4a is a potent inhibitor of antiviral stress response (Menachery et al., 2017; Rabouw et al., 2016). Together with ORF4a, accessory proteins ORF4b, ORF5, and structural proteins M and N are potent inhibitors of interferon (Yang et al., 2013; Chang et al., 2020).

The severe disease manifestation seen following infection with MERS, SARS-CoV, and SARS-CoV-2 could be explained by massive cytokine storm triggered by viruses. All three viruses infect airway epithelial cells, where they replicate. MERS-CoV, besides airway epithelial cells, was also detected within human T cells where it was shown to cause apoptosis (Chu et al., 2016). Such ability to infect and cause apoptosis of T cells is not observed in other human coronaviruses, and can contribute to the difference seen in MERS-CoV pathogenesis. As reported by Liu et al. SARS-CoV-2, SARS-CoV and MERS-CoV could infect dendritic cells, mononuclear macrophages and other peripheral blood mononuclear cells, inducing the cells to release large amounts of cytokines and chemokines (Liu et al., 2021; Zhou et al., 2015). Together with infection of dendritic cells, mononuclear macrophages and other peripheral blood mononuclear cells, MERS-CoV virus was suggested to be replicated in them, further causing apoptosis and abnormal cytokine release from infected cells (Lau et al., 2013). This sets MERS-CoV virus apart from other coronaviruses. In summary, it is possible that MERS-CoV’s higher mortality are due to unique properties of the virus, such as replication in immune cells, leading to their apoptosis, delayed interferon response, and abnormal cytokine release. High levels of IL-6, IP-10, IL-8, RANTES, and IFN-α were detected in the serum of patients with severe MERS compared to those with mild MERS (Min et al., 2016; Kim et al., 2016). Serological studies of patients with MERS-CoV have shown that low levels of IFN-alpha secretion were observed in patients with severe disease up to death, while low levels of type I IFN correlate with recovery and positive outcome (Faure et al., 2014). Factors such as the time interval between the onset of symptoms and hospitalization, age, and comorbidities influence the further course of the disease, up to and including a fatal cytokine storm (Ahmadzadeh et al., 2020).

Overall, the differences in the viral infection outcome are due to difference in viruses’ structure and function. This review summarizes similarities and highlights differences in the structure and function of structural proteins in all seven human coronaviruses.

The genome of all seven coronaviruses is similar in organization. It contains coding regions for four structural proteins (spike glycoprotein (S), nucleocapsid phosphoprotein (N), membrane protein (M), and envelope (E) protein), 15 non-structural proteins (nsp) and 7 accessory proteins (Brant et al., 2021). The detailed summary of structural features of four structural proteins is provided along with their main functions in viral lifecycle as well as in host cell physiology.

## 2 Structure and function of S protein

### 2.1 Structure

For all seven types of human coronaviruses, the spike protein is a key structural element that plays a crucial role in viral infection. S proteins form large crown–like spikes on the surface of the virus, the feature that gave the name of the taxonomic group of viruses - coronaviruses. In coronaviruses, including SARS-CoV-2, SARS-CoV, MERS-CoV, HCoV-HKU1, HCoV-OC43, HCoV-NL63 and HCoV-229E, the spike protein acts as a type I transmembrane fusion glycoprotein. During viral entry, the spike protein interacts with the protein receptors of the host cell and is split into two functional subunits: S1 and S2. Depending on the coronavirus type, the S1/S2 spike protein site is cleaved either by furin in the infected cell or by host cell proteases during viral entry (Kirchdoerfer et al., 2016; Hoffmann et al., 2020; Shang et al., 2020). The presence of multiple arginine residues at the S1/S2 site is believed to make the protein more susceptible to cleavage. Besides SARS-CoV-2, three (MERS-CoV, HCoV-OC43 and HCoV-HKU1) of the six other HCoVs have furin cleavage sites (Liu et al., 2021). Within SARS-CoV-2 virus the furin-cleavage site is distinguished by four amino acids: 681-PRRA-684. Knockout of furin with CRISPR-Cas9 showed a significant reduction in cleavage in the S1/S2 region of SARS-CoV-2 spike glycoprotein, but did not completely prevent this process (Wrapp et al., 2020; Papa et al., 2021; Ou et al., 2020). *In vitro* studies of the S1/S2 region of the SARS-CoV-2 spike protein highlighted the importance of residue R683 in the RRAR motif for furin recognition. Besides this, serines at the edges of this motif, specifically S680 and S686, can be phosphorylated by basophilic and proline-directed kinases, which negatively affects furin cleavage at this site (Örd et al., 2020). Substituting proline with arginine at residue 681 disrupts the proline motif necessary for phosphorylation, which in turn increases the potential for furin cleavage at this site (Beaudoin et al., 2022).

It is worth mentioning here that MERS-CoV virus is a unique in its structure compared to other human coronaviruses as far as it has two furin-recognition motifs (RXXR): at S1/S2 border and at S2’ site. This factor was suggested to contribute to high pathogenicity of MERS-CoV (Millet and Whittaker, 2014; Wu and Zhao, 2020).

As far as S protein has to be proteolytically activated in order to be able to fuse with host cell membrane, it is of interest to focus on proteases that activate coronaviruses without furin-recognition site. The SARS-CoV S protein contains several sites that are subject to cleavage, but the major furin cleavage site has not been identified. For SARS-CoV it was shown that endosomal cysteine protease – cathepsin L plays role in membrane fusion (Simmons et al., 2005). Besides cathepsin L, other proteases, such as elastase and coagulation factor Xa were shown to be active on SARS-CoV (Matsuyama et al., 2005; Du et al., 2007). The furin cleavage site is absent in HCoV-229E, while for HCoV-NL63 it is located in S2’ site (Wu and Zhao, 2020). Human coronavirus 229E uses various proteases, such as cathepsin L, TMPRSS2, and trypsin, to activate cell entry (Kawase et al., 2009). The I577S mutation in the spike protein allowed increased cathepsin utilization by the 229E virus, however, reduced cell replication ability was observed. This may indicate that the endosomal route of coronavirus entry is less preferable for 229E compared to activation via TMPRSS2 (Shirato et al., 2017; Bonnin et al., 2018).

Interestingly, peptides with amino-acid sequences which are similar to the furin cleavage-sites of original SARS-CoV-2 virus as well as its variants (Alpha, Delta, and Omicron) are potent inhibitors of α7 and α9α10 nicotinic acetylcholine receptors (Hone et al., 2024). Considering that both α7 and α9α10 receptors are abundantly expressed in lung epithelial cells (Hollenhorst and Krasteva-Christ, 2021), it could be hypothesized that the receptors are used by SARS-CoV-2 as an alternative to ACE2 entry point to the host cell. α7 and α9α10 receptors are also expressed by immune cells, where they modulate cytokine release (Fujii et al., 2017). It is possible that these receptors contribute to the immune response observed in SARS-CoV-2 infection.

**Figure 1:**
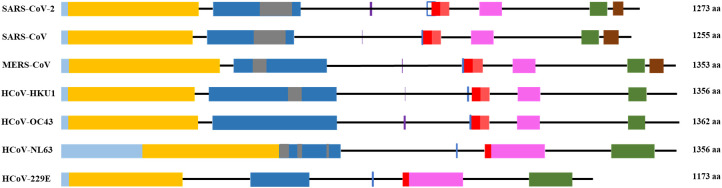
General scheme of the S protein structure. Following color code is used: light blue- signal sequence, orange - N-terminus domain of S1 subunit, blue – C-terminus domain of S1 subunit, grey – receptor binding motif, red – fusion peptide, pink – heptad repeat 1, green – heptad repeat 2, brown – transmembrane domain. Purple vertical line - S1/S2 cleavage site, blue vertical line - S2’ cleavage site.

The S1 subunit is responsible for primary contact, while the highly conserved S2 subunit promotes membrane fusion between the host cell and the virus (Heald-Sargent and Gallagher, 2012; Holmes et al, 1981; Perlman and Netland, 2009; Künkel and Herrler, 1993; Qian et al., 2013). The S1 subunit contains the N-terminal domain (NTD), and C-terminal domain (CTD), harboring receptor-binding domain (RBD) and receptor-binding motif (RBM) (Peng et al., 2021) in all but HCoV-OC43 virus (**Figure 1** and **Supplementary Table 1**). According to Caetano-Anolles et al. the NTD is characterized by a galectin-like structure, a common target of mutations, which helps the virus evade the physiological responses of the host (Guruprasad, 2021; Caetano-Anollés et al., 2022).

Mutations within S protein change the affinities of the RBD to the host receptor, and lead to emergence of new viral variants with increased infectivity and transmissibility. There are five variants of concern of SARS-CoV-2 that emerged during the COVID-19 pandemic: Alpha (B.1.1.7), Beta (B.1.351), Gamma (P.1), Delta (B.1.617.2) and Omicron (B.1.1.529). The alpha variant of SARS-CoV-2 has 23 mutations, 9 of which are within S protein. Mutations that change the biological processes of the virus include N501Y (increases binding to the target receptor), P681H (improves transmissibility) and D614G (Liu et al., 2022; Mohammadi et al., 2021; Yang et al., 2021). D614G mutation in the wild type of SARS-CoV-2 increased the infectivity and transmissibility of the virus (Daniloski et al., 2021). Also, the S1/S2 junction cleavability has been increased by the D614G mutation (Gobeil et al., 2021). The beta variant has three mutations in the RBD, these include K417N, E484K and N501Y, and six mutations in the remaining regions of the spike. Similarly to the N501Y alpha variant, the K417N and E484 substitutions improve the affinity of the virus for its target receptor, angiotensin-converting enzyme 2 (ACE2) (Funk et al., 2021). The gamma variant of SARS-CoV-2 has 17 nonsynonymous mutations, ten of which are located in the S protein genes. Among them K417T, E484K and N501Y mutations which help the virus to escape immune surveillance (Chen et al., 2021; Faria et al., 2021; Naveca et al., 2021; Wang et al., 2021). In the delta variant of SARS-CoV-2, 17 mutations were found. In S1, two deletions occurred: E156del and F157del (Chen et al., 2020; Tatsi et al., 2021). The P681R mutation increases the cleavability of the S1/S2 site, which leads to increased replication in the delta variant of SARS-CoV-2 (Takeda, 2022; Saito et al., 2022). The F306L mutation of the SARS-CoV-2 delta variant may lead to strengthened binding between ACE2 and spike protein, and this mutation has been reported to be associated with increased mortality during infection (Sila et al., 2024). In the omicron variant, 39 mutations were identified in the spike protein, 15 of which were found in the RBD region. The mutations Q498R and Q493K were reported to weaken the RBD-ACE2 interaction, while P681H resulted in high transmissibility of the virus (Kim et al., 2021).

Similarly to SARS-CoV-2, S protein of other human coronaviruses was shown to have mutations that influence the pathogenicity of the viruses. For example, using next generation sequencing, a subgenotype of the HCoV NL63 virus with the I507L mutation was identified, which enhanced the penetration of the virus into the host cells, and was associated with severe course of the lower respiratory tract disease (Wang et al., 2020). Point mutations H183R and Y241H in the spike protein of the HCoV-OC43 virus resulted in weakened protein synthesis and increased neuroinvasiveness of the virus, which caused apoptosis in mouse neuronal cells (Favreau et al., 2009).

The amino acid sequence of the S protein and RBD of SARS-CoV compared to SARS-CoV-2 is 76% and 74% identical, respectively (Jaimes et al., 2020). The RBD of the SARS-CoV-2 S protein is located between 319 and 541 residues. The SARS-CoV RBD is located between residues 306 and 526 with residues. It forms an extended tyrosine-rich loop and binds directly to the angiotensin-converting enzyme 2 (ACE2) receptor (Li et al., 2005). According to Li and colleagues, the region spanning 577-597 residues of the SARS-CoV RBD matches the S1 region of HCoV-NL63 (Li et al., 2007).

The RBD has a core subdomain characterized by a five-stranded antiparallel β-sheet (found in β-HCoV) or a six-stranded β-sandwich (found in α-HCoV) and a receptor-binding subdomain. The differences in the receptor-binding subdomain explain the affinities of the virus to the target receptor. For example, MERS-CoV has unique features within its receptor-binding subdomain, enabling it to bind to DPP4. Particularly, unlike SARS-CoV and SARS-CoV-2 which have extended loop between two short antiparallel β-strands, MERS-CoV has a β-sheet structure made up by four antiparallel β-strands (Wang et al., 2013).

In almost all HCoVs, the RBD was found in the CTD of the S1 subunit. Only OC43 is distinguished by the location of the RBD in the NTD. It is also known that the S1-NTD of the HCoV-HKU1 virus mediates primary attachment via glycan binding (Qian et al., 2015; Kirchdoerfer et al., 2016).

Following interaction of the RBD with the cell receptor, viral entry into the host cell requires the use of proteases to activate the spike protein (Bestle et al., 2020; Hoffmann et al., 2020).

The S2 subunit of the spike protein is highly conserved compared to the S1 subunit. S2 consists of fusion peptide (FP), heptad repeat 1 (HR1), heptad repeat 2 (HR2) and transmembrane domain (**Figure 1**). Interaction of the virus with the host cell leads to refolding of HR1 and releasing of the fusion peptide. FP mediates the fusion of the host cell membrane lipid bilayer with the viral membrane and further cell hijacking (Zhang et al., 2018; Heald-Sargent and Gallagher, 2012; Belouzard et al., 2012; Li, 2016; Huang et al., 2020; Xia et al., 2020). The comparative structures of human coronaviruses are presented in the **Table 1**.

**Table 1:** Structural comparison of S protein from different coronaviruses, top and side views.

Coronavirus	Top view	Side view	PDB	UniProt	Ref.
SARS-CoV-2	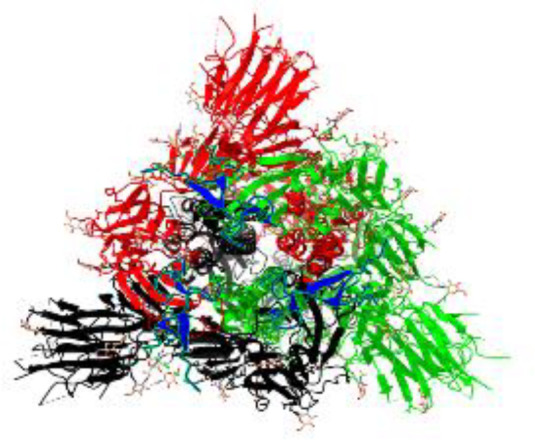	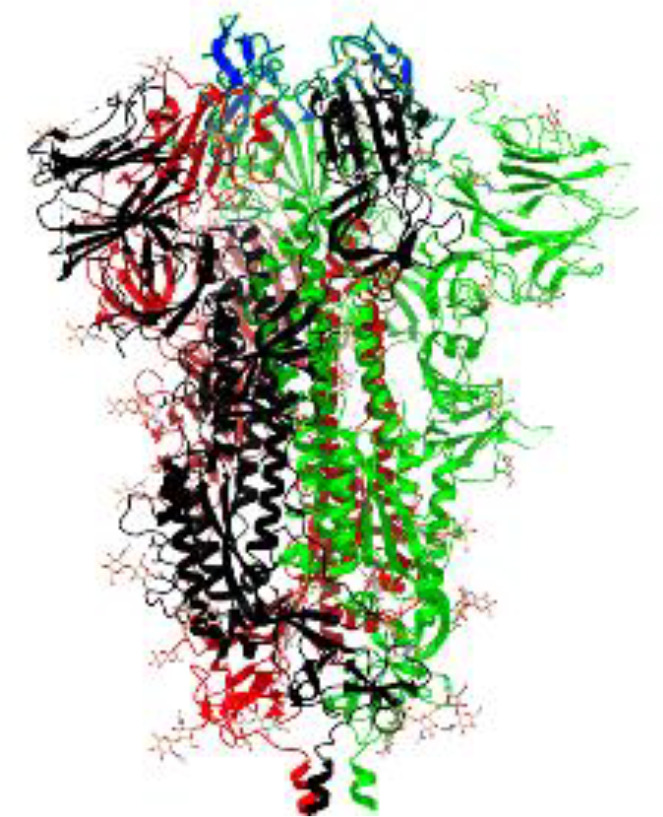	6VXX	P0DTC2	(Walls et al., 2020)
SARS-CoV	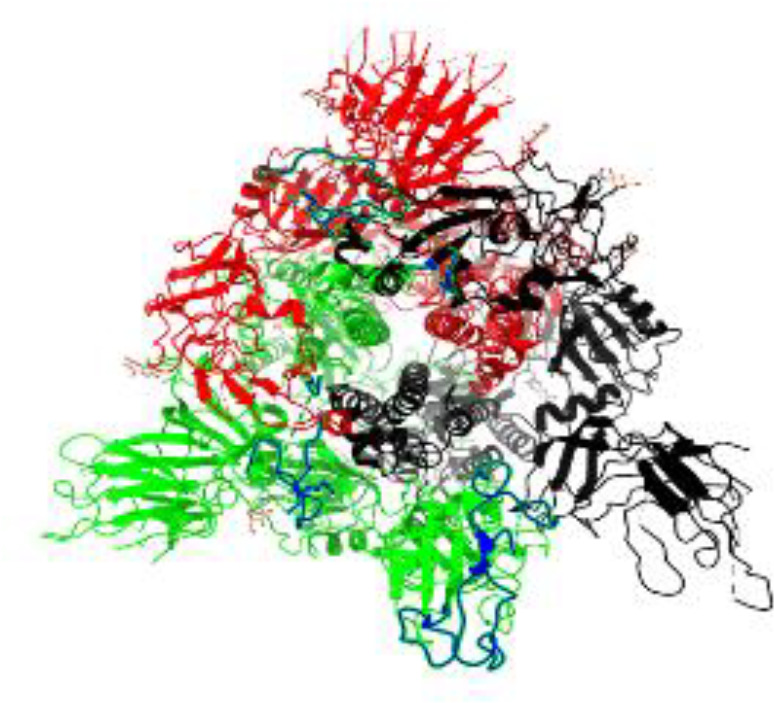	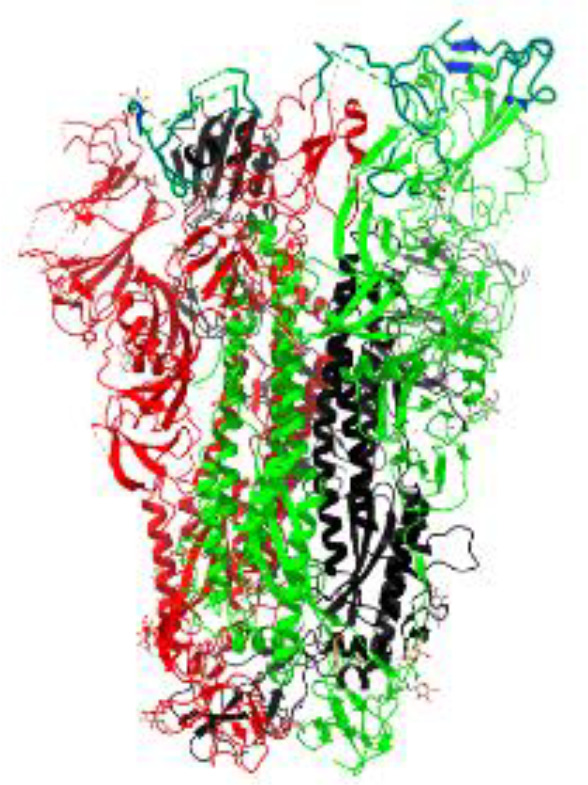	7ZH5	P59594	(Toelzer et al., n.d.)
MERS-CoV	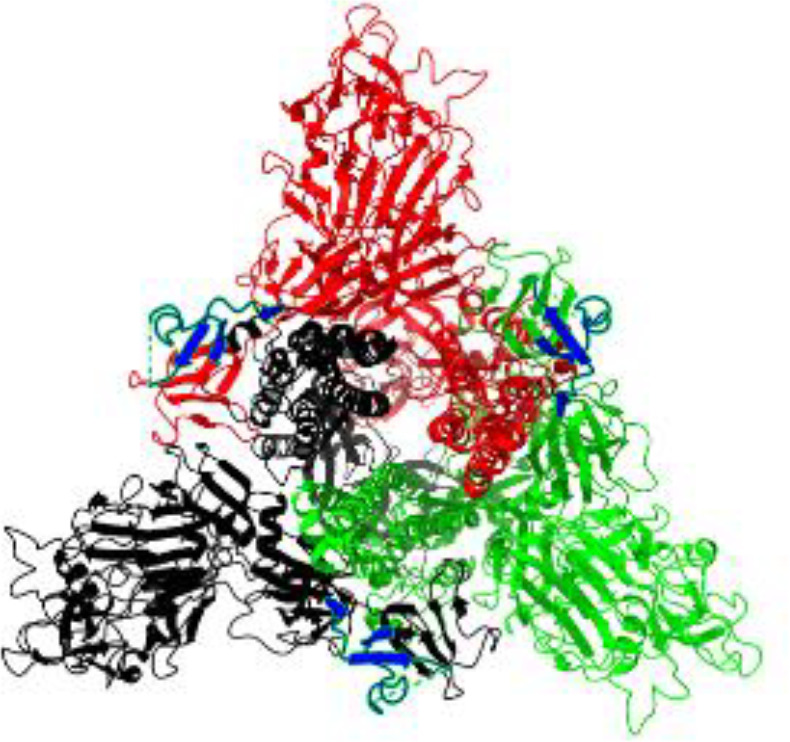	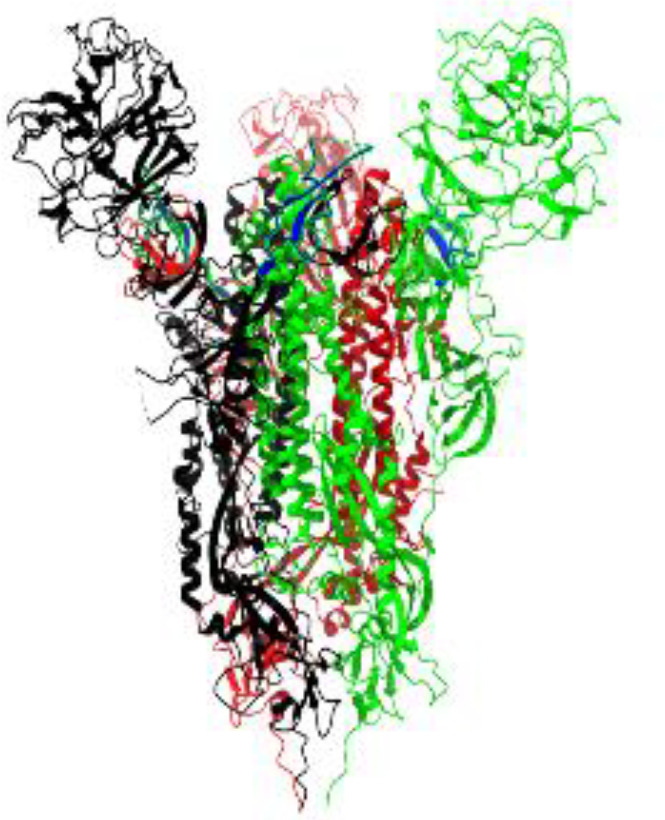	7X27	K9N5Q8	(Zhang et al., 2022)
HCoV-HKU1	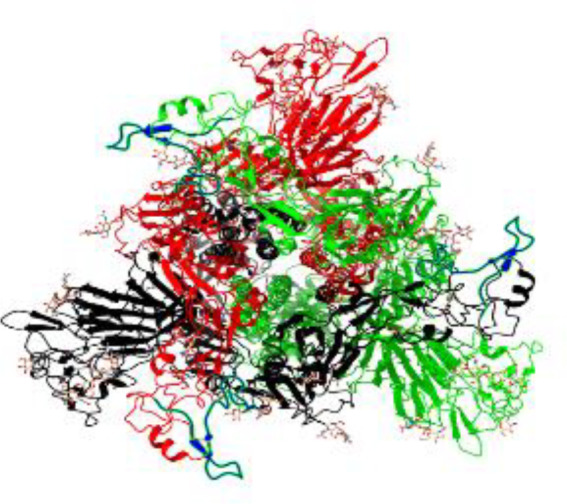	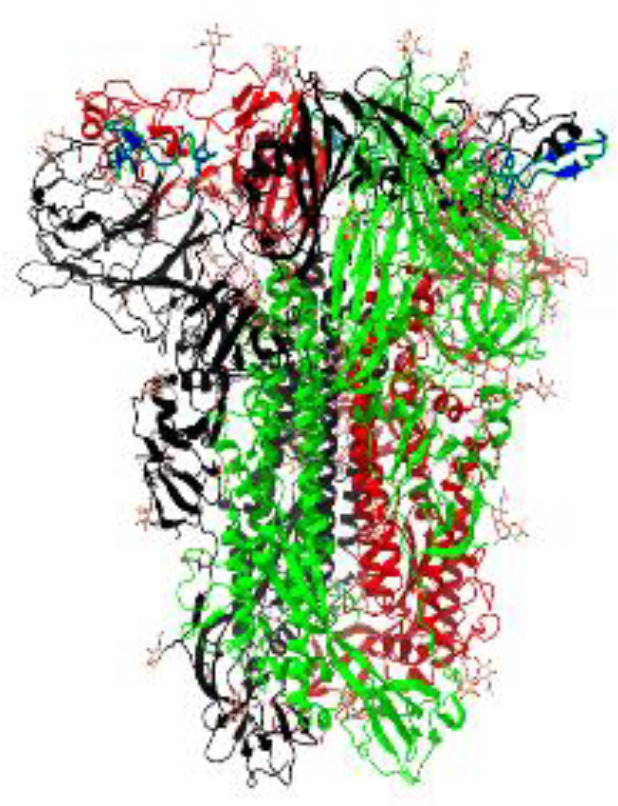	8OHN	Q5MQD0	(Pronker et al., 2023)
HCoV-OC43	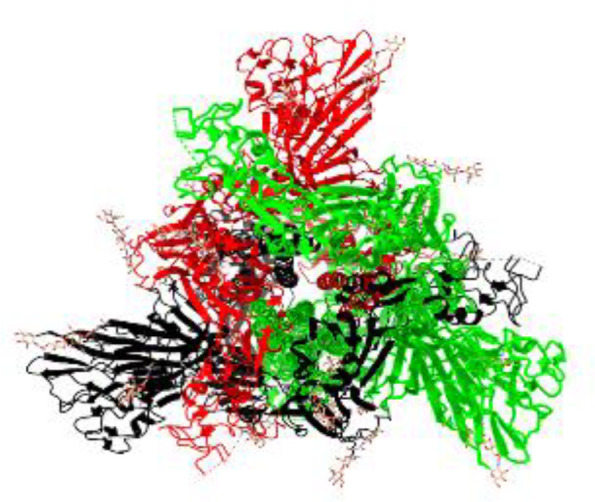	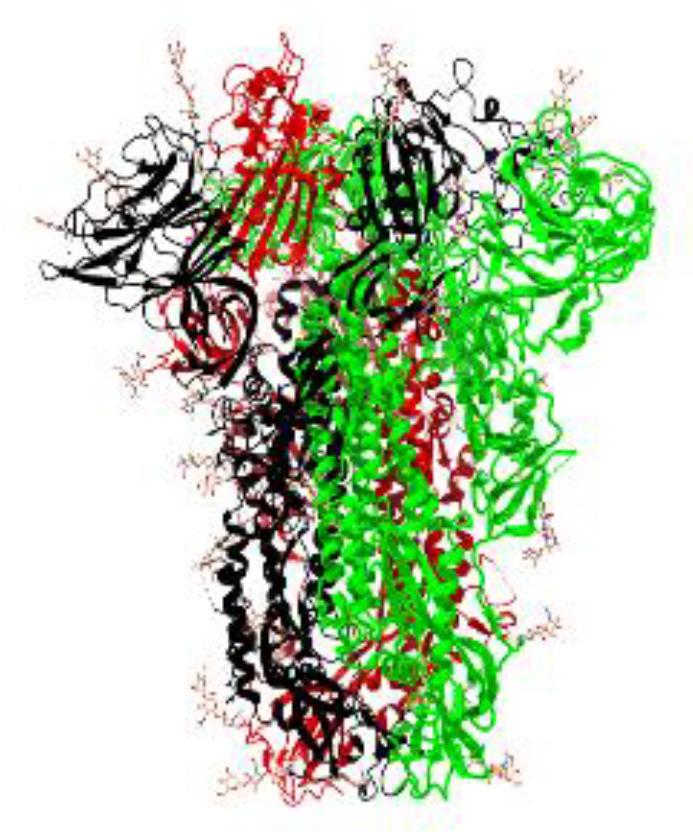	6OHW	Q696P8	(Tortorici et al., 2019)
HCoV-NL63	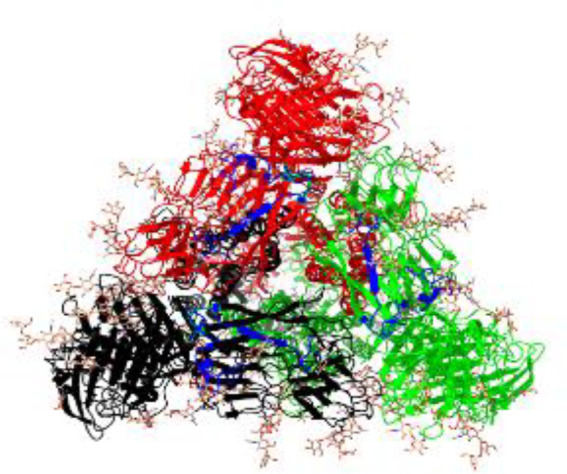	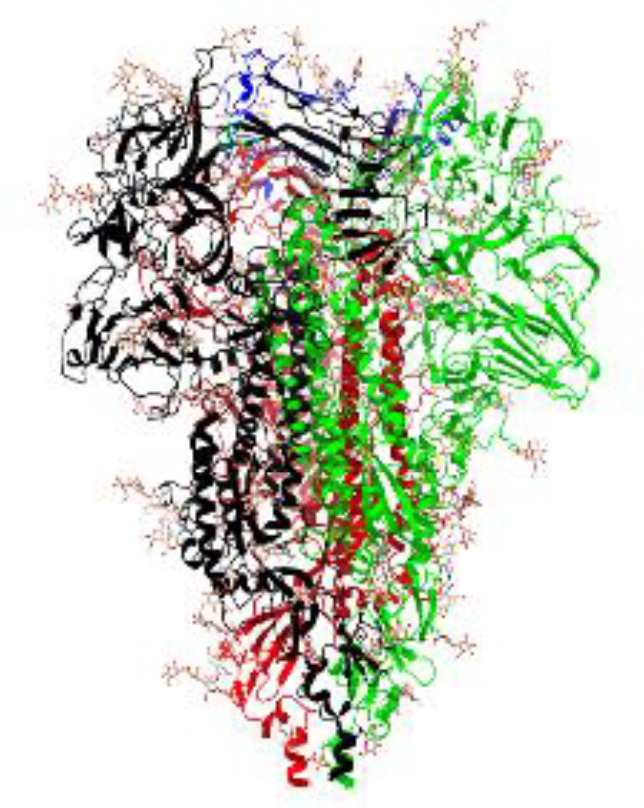	7KIP	Q6Q1S2	(Zhang et al., 2020)
HCoV-229E	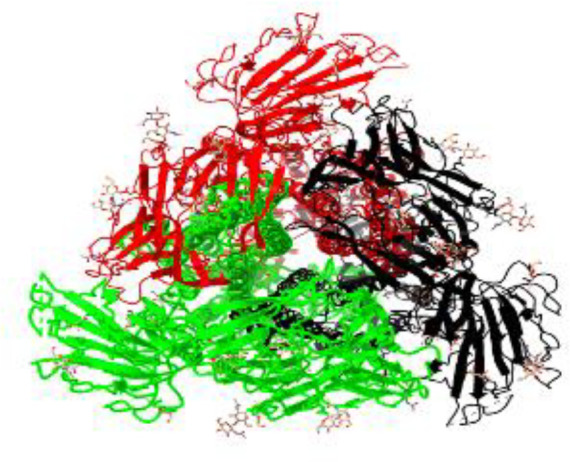	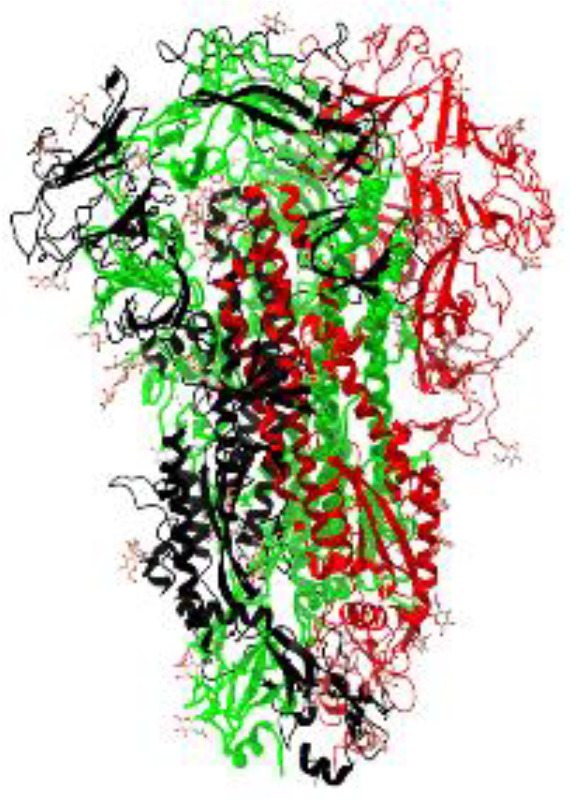	7CYC	P15423	(Song et al., 2021)

Monomers are in red, black, and green, receptor binding motif is in blue.

According to the data provided by Song F. et al., using the pseudotyping method with Modified Vaccinia virus Ankara, it was determined that the full-length S protein of MERS-CoV, in its N-glycosylated state, is estimated to be 210 kDa. Subsequent investigations propose the cleavage of the mature full-length S glycoprotein into an amino-terminal domain (S1) and an approximately 85-kDa carboxy-terminal domain (S2), which is membrane-anchored. The cleavage occurs between 751 and 752 residues (Song et al., 2013).

SARS-CoV-2, SARS-CoV and MERS-CoV exhibit structural mimicry with several alternative receptors aside from primary ones. For example, all three have similar receptor binding motifs (RBMs) to complement Factor H and EGF-like domains. SARS-CoV showed mimicry for clitocypin-5 cysteine protease, von Willebrand factor, and intracellular adhesion molecule 5. The RBM of SARS-CoV-2 mimics TNF receptors, neuroserpin, IL-6 receptors, and ephrin-B2. MERS-CoV mimics TNF ligands, fibronectin type III, transferrin receptor protein 1, and toxoplasma gondii surface antigen 3. These structural resemblances imply possibility for alternative pathways through which coronaviruses could modulate host cell invasion, cellular metabolism, immune responses, and disease severity (Beaudoin et al., 2021).

The HCoV-HKU1 and HCoV-OC43 spike glycoproteins consist of 1356 and 1362 amino acids. Similarly to HCoV-OC43, HCoV-HKU1 utilizes 9-O-Acetylated-sialic acid as a receptor to engage the host cells and initiate infection (Millet and Whittaker, 2014; Huang et al., 2015; Li et al., 2020).

HCoV-NL63 and HCoV-229E belong to the alpha subgroup of coronaviruses. The HCoV-NL63 spike protein belongs to type I single-chain transmembrane glycoprotein, the molecular weight of which is estimated at 128-160 kDa before and 150-200 kDa after glycosylation. Comparison of the amino acid sequence of HCoV-NL63 with HCoV-229E, SARS-CoV-2 and SARS-CoV showed 50%, 17.1%, and 25% sequence identity, respectively. HCoV-NL63 shares ACE2 receptor binding with SARS-CoV-2 and SARS-CoV, and uses ACE2 as a target receptor required for cellular entry (Hofmann et al., 2005). A distinctive feature of HCoV-NL63 is that the N-terminus region of the spike protein contains 179 amino acids that have no homology with any of the HCoVs (Smith et al., 2006; Castillo et al., 2023; Pöhlmann et al., 2006; Brielle et al., 2020).

HCoV-229E is known to cause mild respiratory infections in humans (Hamre and Procknow, 1966). The specific receptor for HCoV-229E is aminopeptidase N (APN), also known as CD13. The spike protein binds to the APN receptor, initiating the attachment and fusion of the virus with the host cell membrane (Yeager et al., 1992). The findings by Blau et al. suggest that HCoV-229E undergoes endocytosis following the binding of the spike protein at the plasma membrane. Subsequently, the virion is sorted into endosomes, where fusion between the viral envelope and endocytic membrane takes place (Blau and Holmes, 2001).

### 2.2 Function

#### 2.2.1 Cellular entry

The most crucial function of spike proteins is the facilitation of cellular entry into the host cell via receptor binding and fusion (Pillay, 2020). The trimeric protein in SARS-CoV-2 viruses accomplishes that by binding to angiotensin-converting enzyme 2 of the host cell through the receptor binding domain within the S1 subunit. S protein of SARS-CoV-2 and MERS-CoV is cleaved into S1 and S2 proteins during viral biosynthesis in a host cell, then S2 protein is cleaved at S2’ site. While S1 binds the receptor, the S2 subunit acts as an anchor of the S protein to the membrane of the virus and facilitates membrane fusion.

**Figure 2:**
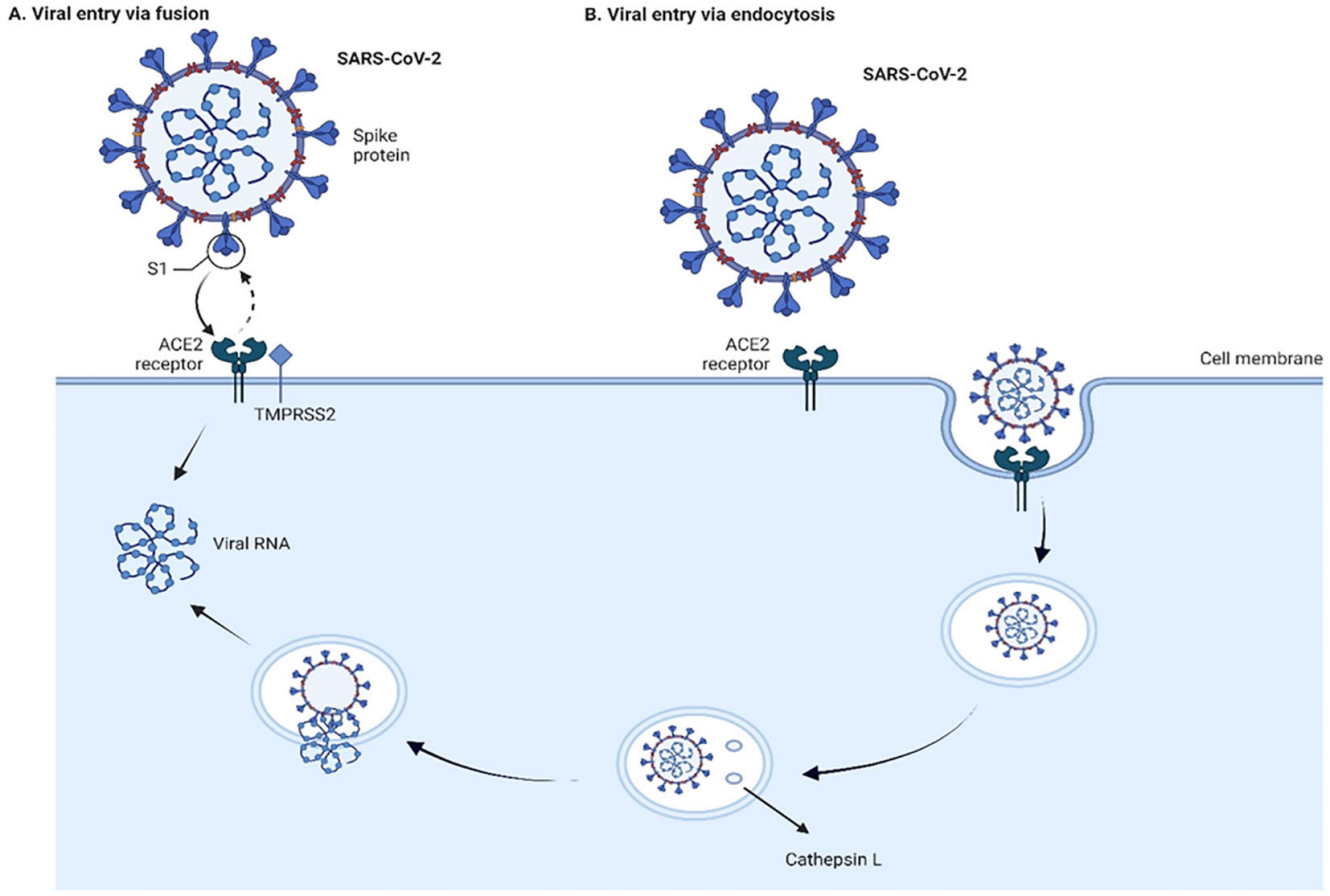
Viral entry into the cells through fusion **(A)** and endocytosis **(B)** Two pathways of cell entry are shown: endosomal and through fusion. The endosomal route is mediated in the case of a deficiency of transmembrane protease, serine 2 (TMPRSS2), and the virus-ACE2 complex internalizes. It does so through clathrin-mediated endocytosis, and the cleavage takes place inside the endosome via cathepsin L which requires a low pH environment. In the case of sufficient amount of TMPRSS2, the cleavage is done on the surface of the host cell membrane by TMPRSS2. The figure is created using BioRender.

SARS-CoV and SARS-CoV-2 can enter cells through non-endosomal and endosomal routes (Hofmann and Pöhlmann, 2004; Peng et al., 2021; Cesar-Silva et al., 2022; Simmons et al., 2005) (**Figure 2**). Non-endosomal route involves fusion of viral envelope with host cell membrane. The process of initiation of virion entry to the host cell occurs by binding of the spike protein to a receptor on the cell surface. The endosomal route relies on clathrin-mediated endocytosis. When compared to non-endosomal entry, endocytosis leaves no traces of viral proteins on the membrane, which allows the virus to evade detection by immune cells.

Proteolytic cleavages of S protein are essential for binding to ACE2. Cleavages at the S1-S2 boundary are a requirement for virus maturation, they occur in the trans-Golgi network of the viral-producer host cell, and are carried out by Ca^2+^-dependent proprotein convertase – furin (Johnson et al., 2020). Cleavage on S2’ site occurs during the viral entry, and it is associated with target-cell proteases. Depending on the type of viral entry pathway, two major proteases are involved in the cleavage: cathepsin L in endosomal entry pathway and transmembrane protease, serine 2 (TMPRSS2) in the cell surface entry pathway (**Figure 2**). Inhibition of furin or/and TMPRSS2 results in deactivation of the S protein (Shapira et al., 2022; Essalmani et al., 2022).

The engagement of spike protein with ACE2 for viral fusion and entry is a complex multistep process. First, the RBD changes its conformation to a slightly open state that allows binding to the receptor. This in return exposes S2’ site as the protein refolds and HR1 thrusts the cell membrane which inserts FP. The following dissociation of S1 causes the folding back of HR2 that leads to the juxtaposition of FP to the transmembrane region and the fusion of the membranes. The process continues with the formation of the fusion pore by the same HR2 leading to the facilitation of viral entry (Jackson et al., 2022).

ACE2 is the main cellular receptor for coronaviruses SARS-CoV-2, SARS-CoV, and HCoV-NL63 (Hofmann et al., 2005; Jackson et al., 2022; Beyerstedt et al., . 2021; Li et al., 2007). It has been found that early trypsin priming may enhance SARS-CoV-2 infection in cultured cells (Kim et al., 2022). Similarly, an increase in the infectivity of SARS-CoV is seen in the presence of proteases such as trypsin, elastase and thermolysin (Matsuyama et al., 2005). Various receptor molecules are also known to facilitate the penetration and infection of the SARS-CoV-2 virus (Alipoor and Mirsaeidi, 2022; Mollentze et al., 2022; Xia, 2023), such as neuropilin receptors (Daly et al., 2020; Cantuti-Castelvetri et al., 2020), C-lectin type receptors, dendritic cell-specific intercellular adhesion molecule-3-grabbing non-integrin, Liver/lymph node-specific intercellular adhesion molecule-3-grabbing integrin, Macrophage Galactose-type Lectin (Thépaut et al., 2021; Lempp et al., 2021), glucose-regulated protein (Carlos et al., 2021; Shin et al., 2022), heparan sulfate proteoglycans (Zhang et al., 2023; Kearns et al., 2022; Bermejo-Jambrina et al., 2021), AXL Receptor Tyrosine Kinase (Wang et al., 2021). Another receptor molecule that also facilitates viral entry is CD147. Ragotte RJ et al. showed that CD147-mediated facilitation is not via binding to the RBD region of the virus (Ragotte et al., 2021).

The principle of the viral entry in MERS-CoV is similar in terms of the process to the SARS-CoV. However, a major distinct feature is a different receptor used by the virus for attachment and entry. Human dipeptidyl peptidase 4, type II transmembrane ectopeptidase serves as a receptor for MERS-CoV (Meyerholz et al., 2016). Depending on the tissue type and host cell, entry of MERS-CoV into cells can occur either through fusion or via endosomes. Experiments by Qian et al. carried out on VERO E6 and 293T cell lines show that if the MERS-CoV spike protein on pseudovirions is not digested by trypsin or TMPRSS2/4 proteases, then the viruses enter through endocytosis in a cathepsin L-dependent manner. However, if the MERS-CoV S protein is cleaved either during virus maturation by proteases or by trypsin in the extracellular fluid, the viruses penetrate the plasma membrane at neutral pH. This induces syncytia formation even in cells that express low or no levels of the MERS-CoV receptor (Qian et al., 2013).

HCoV-OC43 utilizes sialic acids, specifically N-acetyl-9-O-acetylneuraminic acid as an attachment receptor to bind to the host cell surface. This is similar to the bovine coronavirus, suggesting zoonotic origins for OC43. Unlike many other human coronaviruses which employ receptors like aminopeptidase N or angiotensin-converting enzyme 2, OC43 was found to use either HLA class I molecules or sialic acids as its fusion receptor for cellular entry.

Upon receptor binding, OC43 initiates a caveolin-mediated endocytic pathway for internalization. The virus particles go to caveolae, which are flask-shaped invaginations in the cell membrane containing caveolin-1. This caveolar route allows OC43 cellular entry in a manner distinct from other coronaviruses that primarily use clathrin-mediated endocytosis. While actin filaments are not directly required, unwinding of the actin cortex at the cell surface seems necessary to facilitate OC43 receptor binding and initial entry via caveolae-mediated endocytosis (Owczarek et al., 2018).

Similar to OC43, HCoV-HKU1 uses sialic acids for binding to the surface membrane of the host cell (Liu et al., 2021). However, Saunders et al. (2023) showed that TMPRSS2 acts as a receptor for HCoV-HKU1, facilitating viral entry in two ways. First, its enzymatic activity primes the virus for membrane fusion at the cell surface. Second, even when TMPRSS2 is inactive, it can still bind the virus, allowing entry through endosomes. This dual role suggests TMPRSS2 is crucial for HKU1 entry and a potential target for antiviral strategies (Saunders et al., 2023).

The most probable receptor for HCoV-229E is human aminopeptidase N (hAPN) which is identical to CD13, a glycoprotein of monocytes, granulocytes and their progenitors (Yeager et al., 1992).

#### 2.2.2 Cellular effects of the S protein

The numerous studies show that spike protein initiate ER stress induction and activation of unfolded protein response, which in turn leads to innate immune response, microRNA modulation, autophagy and cell death (Xue and Feng, 2021; Versteeg et al., 2007; Chan et al., 2006).

It was investigated whether the SARS-CoV-2 spike protein could prime the NLRP3 inflammasome in microglia cells, in addition to directly activating it. The cells were exposed to the spike protein, and induction of priming of inflammasomes through NF-κB signaling was observed. Priming would make the cells more responsive to subsequent inflammasome triggers. When ATP or nigericin were added following spike protein exposure, higher IL-1β release was observed compared to the control without prior spike protein exposure. This showed that spike protein indeed induces inflammasome activation in microglial cells through NF-κB (Albornoz et al., 2023).

SARS-CoV-2 S protein engagement with the ACE2 receptor reduces the expression of ACE2 on the cell over time. It also can induce caspase activation with following apoptosis in endothelial cells. Spike protein reduces the production of KLF2 (Krüppel-like Factor 2) and increases the expression of vWF (von Willebrand factor) in primary human arterial endothelial cells. This in turn leads to vascular inflammation and coagulation due to endothelial cell dysfunction (Panigrahi et al., 2021). In HEK293 cells presence of spike protein results in syncytia formation and cell sloughing. The protein also induced TNF-α, MCP-1, and ICAM1 mRNA expression as well as of heme oxygenase-1 (Singh et al., 2022). It was also observed that S protein’s RBD of SARS-CoV-2 upregulates secretion of the IL-6 and IL-8 through ATP/P2Y2 and ERK1/2 signaling pathways in human bronchial epithelia (Zhang et al., 2024). Recombinant subunits of spike protein of SARS-CoV-2 contrary to the previous studies induce CXCL10 chemokine expression that is attenuated via glycogen synthase kinase-3 inhibitor, not through the NF-kB, but rather IRF transcription factor that is TLR2-independent in human macrophage cells (THP-1) (Ghazanfari et al., 2024). Through the Proliferating Cell Nuclear Antigen (PCNA) expression it was identified that S protein of SARS-CoV-2 suppresses cell proliferation of SiHa cell line. The significant increase of expression of anti-proliferative p53 molecule is a suggested mechanism for cellular apoptosis along with pro-apoptotic TRAIL (TNF-related apoptosis-inducing ligand) that is also upregulated (Willson et al., 2024).

The findings of recent research by Monaco et al. (2024) suggest that the S1 domain of spike protein inhibits lactate dehydrogenase B which converts lactate to pyruvate through depletion of NAD+. Such inhibition shifts the metabolism from aerobic to anaerobic pathways. This shift is similar to the Warburg Effect, observed in viral infections and cancers, with cells relying more on glycolysis despite abundance of oxygen. Along with that, the upregulation of proteins contributing to Warburg Effect such as hexokinase-2 (HK2), hypoxia up-regulated protein 1(HYOU1), and TBC1 domain family member 4 (TBC1D4) in the HEK-293T transfected cell line with S1 domain of the S protein was observed in comparison to the control (Monaco et al., 2024).

Another novel finding regarding alternative binding of S protein to β1- and β2-ARs in cardiomyocytes suggests that the virus contributes to cardiac dysfunction which is observed in post-acute sequelae cardiovascular syndrome (PASC-CVS) of COVID-19. Activation of those receptors increases cAMP accumulation in the downstream signaling, and leads to cardiac sympathetic hyperactivity and thus weakens heart function (Deng et al., 2024).

Incubated RAW 264.7 macrophages with truncated spike protein of SARS-CoV induced secretion of IL-6 and TNF-α cytokines through NF-κB pathway (Wang et al., 2007).

A single point mutation (Y241H) in the spike protein of HCoV-OC43 was shown to modulate virus-induced neuropathogenesis in mice, resulting in death. Mice infected with the recombinant virus bearing this mutation (rOC/US241) developed a motor paralysis syndrome with demyelination in the spinal cord, while the reference virus caused only encephalitis. rOC/US241 replicated at a similar levels as the reference virus in the brain but persisted longer in the spinal cord. The Y241H mutation led to neuronal dysfunction shown by abnormal neurofilament phosphorylation. It also downregulated the glutamate transporter GLT-1 in astrocytes and strongly activated microglia/macrophages compared to the reference virus. Treatment with an AMPA receptor antagonist reduced motor dysfunction in rOC/US241 infected mice by attenuating neuronal and glial alterations as well as microglial activation (Brison et al., 2011). The effects of the spike protein on a host cell are summarized in the **Table 2**.

**Table 2:** Cellular effects of spike protein of human coronaviruses.

Coronavirus	Cellular effect	Cell Line
SARS-CoV-2	Cellular entry facilitation via ACE2 with additional furin cleavage site (Hoffmann et al., 2020; Ou et al., 2020)	MRC5, 293T, Huh7, A549, HeLa, RS, LLCMK2, Calu3, Vero
	Activates the NLRP3 inflammasome in human microglia (Albornoz et al., 2023)	MDMi
	Through its engagement with the ACE2 receptor, reduces KLF2 expression while increasing vWF, induces endothelial dysfunction, and activates caspases causing apoptosis in primary human aortic endothelial cells (Panigrahi et al., 2021)	Primary human endothelial cells, lung fibroblasts, HAEpCs, CACO-2
	Upregulates TNF-α, MCP-1, and ICAM1, cytoprotective gene HO-1 and relevant signaling pathways (p-Akt, p-STAT3, and p-p38) (Singh et al., 2022)	HEK293, HEK293-ACE2+ (stably overexpressing ACE2), and Vero E6
	The spike protein’s RBD upregulates IL-6 and IL-8 through the ATP/P2Y2 and ERK1/2 signaling pathways ( Zhang et al., 2024)	16HBE14o- (human bronchial epithelial cell line)
	Spike protein subunits induce CXCL10 upregulation that involves a GSK-3 inhibitor and a TLR2-independent mechanism (Ghazanfari et al., 2024)	THP-1 macrophages, PBMC
	Inhibits the growth of cervical cancer cells via upregulation of p53 and pro-apoptotic TRAIL (Willson et al., 2024)	SiHa
	Inhibits lactate dehydrogenase B and depletes NAD+ shifting priority of metabolism from aerobic to anaerobic pathway known as Warburg Effect (Monaco et al., 2024)	HEK-293T, Calu-3, NCM460D, Caco-2, HK-2
	Binds to β1- and β2-adrenergic receptors in heart cells, contributing to the cardiovascular problems seen in long COVID. This interaction boosts cAMP levels, causing receptor overactivity, faster heart rates, increased sympathetic activity, and weakened heart function (Deng et al., 2024)	HEK-293T, NMCMs (Cultured neonatal mouse cardiomyocytes)
SARS-CoV	Cellular entry facilitation via ACE2 (Li et al., 2005)	x-ray diffraction
	Stimulates IL-6 and TNF-α release from RAW264.7 cells; induces NF-κB activation (Wang et al., 2007)	RAW264.7
MERS-CoV	Viral entry through binding to dipeptidyl peptidase IV (DPP4, or human CD26) (Lu et al., 2013)	*in vitro* assay
HCoV-HKU1	Employs glycan-based receptors carrying 9-O-acetylated sialic acid for cellular entry (Hulswit et al., 2019; Huang et al., 2015)	HAE cell culture, HRT18
HCoV-229E	Binds to metalloprotease CD13, and to hAPN (human aminopeptidase N) (Yeager et al., 1992)	Murine NIH3T3 cells transfected with hAPN cDNA
HCoV-NL63	Bind metallopeptidase angiotensin-converting enzyme 2 (Hofmann et al., 2005)	Huh-7, HEK293T
HCoV-OC43	Utilizes HLA class I molecule or sialic acids for cell entry (Owczarek et al., 2018)	HCT-8
	A single Y241H mutation in the spike protein of HCoV- OC43 induced motor paralysis and death in mice by dysregulating neuronal function, glutamate transport and exacerbating glial activation in the spinal cord (Brison et al., 2011)	BHK-21, HRT-18

## 3 Structure and function of E protein

### 3.1 Structure

**Figure 3:**
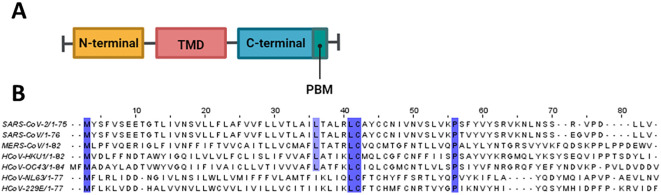
Structural features of E protein. **(A)** Schematic representation of coronavirus E protein structure. **(B)** Multiple sequence alignment was conducted using Clustal Omega and visualized in Jalview. Conserved residues are highlighted in purple.

**Figure 4:**
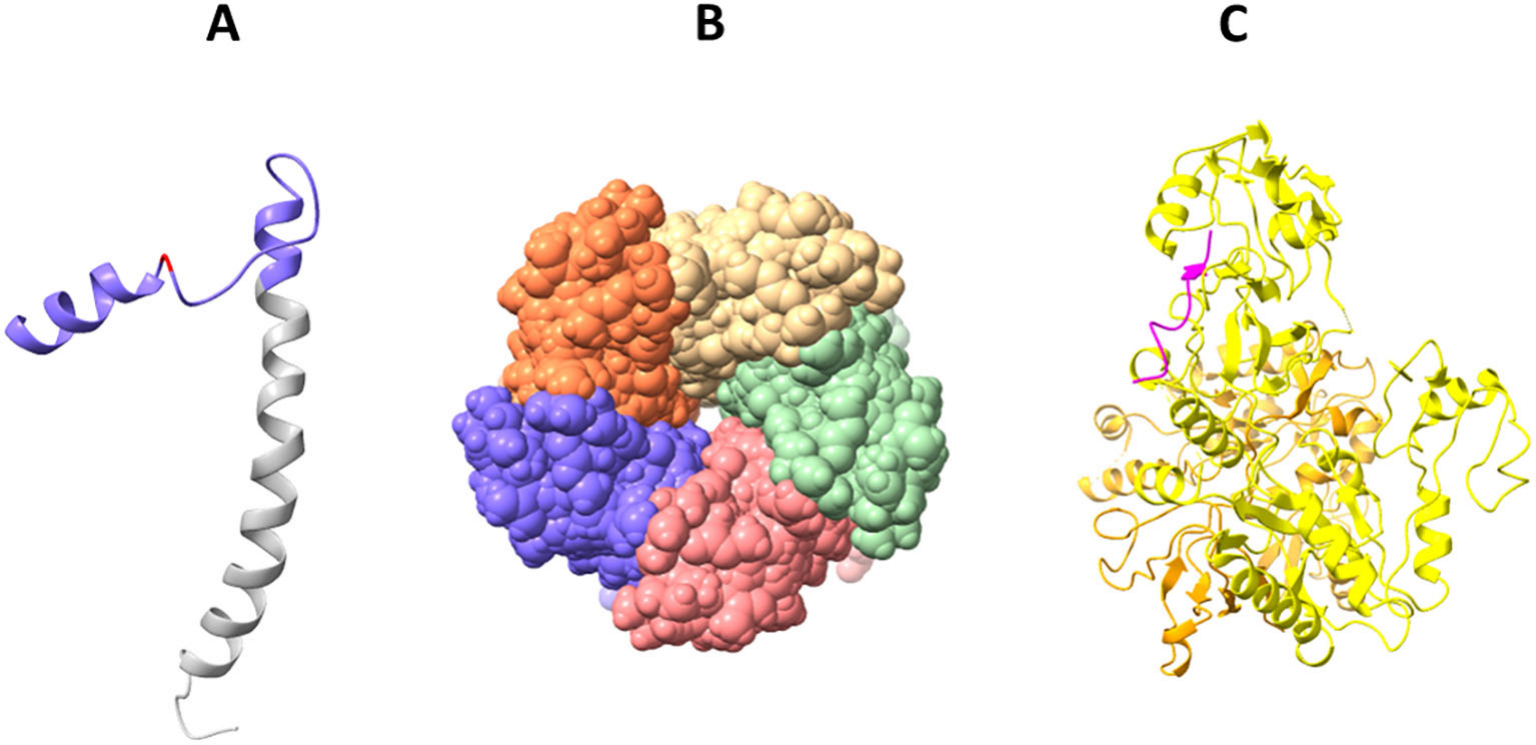
Solved 3D structures of coronavirus E protein. **(A)** SARS-CoV-2 E protein structure. Golgi complex-targeting signal located on the SARS-CoV CTD (colored in purple) is shown in pink, and the TMD is colored in gray (PDB:2MM4); **(B)** Pentameric structure of SARS-CoV-2 E protein transmembrane domain (PDB: 5X29); **(C)** Cryo-electron microscopy structure of SARS-CoV-2 E protein PBM (shown in magenta) interaction with host PALS1 (PDB: 7NTK).

The coronavirus envelope (E) protein is а short, integrаl membrаne protein consisting of 76 to 109 аmino аcids, with а size rаnging from 8.4 to 12 kDа (Schoeman and Fielding, 2019). Structurally, it comprises three distinct domаins, as shown in **Figure 3**. Amino (N)-terminal domain (NTD) is a short, hydrophilic region consisting of 7 to 12 amino acids. Transmembrane domain (TMD) is a large hydrophobic region of 25 amino acids, consisting of at least one predicted amphipathic α-helix. This domain enables the oligomerization of E proteins to form an ion-conductive pore across membranes. Carboxy (C)-terminal domain (CTD) is a hydrophilic region which makes up most of the protein. This domain contains β-coil-β motif, which functions as a Golgi-complex targeting signal (Nieto-Torres et al., 2015) (**Figure 4A**).

The E protein in coronaviruses adopts an N-ecto/C-endo topology with one transmembrane domain. This topology implies that the N-terminus of the protein is located outside the virus (ectodomain), and the C-terminus is located inside the virus (endodomain). This single transmembrane domain is crucial for the integration of the E protein into the host cell membrane (Nieto-Torres et al., 2015; Schoeman and Fielding, 2019).

Solid-state NMR spectroscopy revealed that the transmembrane domain of the E protein of SARS-CoV-2 assembles into a homopentameric structure, forming a narrow pore within membranes (Mandala et al., 2020) (**Figure 4B**). However, the recently conducted NMR study found that it can exist as a dimer in lipid bilayers (Zhang et al., 2023). In addition, an ectodomain-containing E construct (ENTM, aa 1-41) from SARS-CoV-2 forms dimers instead of pentamers in lipid bilayers (Somberg et al., 2024). They found that oligomeric state and drug binding of the E protein is affected by the presence of ectodomain. This research clearly demonstrates that E protein may adopt different oligomeric states, and depending on that the binding of antivirals may be affected. It is therefore hypothesized that E proteins using different oligomeric states perform different functions, which are to be investigated in future studies.

The CTD of the SARS-CoV-2 E protein contains a PDZ-binding motif (PBM), which is crucial for establishing interactions with host cell proteins. This PBM in the CTD of the SARS-CoV-2 E protein interacts with host cell junction proteins such as PALS1 (Chai et al., 2021) (**Figure 4C**). This interaction induces relocation of PALS1 from the cell junction to the endoplasmic reticulum–Golgi intermediate compartment (ERGIC), where viral assembly and maturation take place (Chai et al., 2021). One study found that amino acid variations within the CTD of SARS-CoV-2 E protein, notably at residues Ser 55 -Phe 56, Arg 69, and the C-terminal end (DLLV: 72-75), may alter its binding affinity to PALS1 (Rahman et al., 2021).

Cellular studies showed that SARS-CoV-2 E protein PBM interacts with syntenin and ZO1 (Ávila-Flores et al., 2023). Host cell proteins associated with cellular junction and polarity such as TJP1, PARD3, MLLT4, LNX2 interact with the E protein’s PBM, leading to the sequestration of these PDZ domains to the Golgi compartment (Zhu et al., 2022). All these findings show that the coronavirus uses its E protein to disturb cellular communication and integrity, thereby enabling viral propagation.

Like in SARS-CoV-2, the TM domain of the SARS-CoV E protein forms a pentameric ion channel across membranes. It was shown that leucine and valine rich region within the SARS-CoV E protein TM domain is critical for the formation of the ion channel. Additionally, SARS-CoV E protein possesses a triple cysteine motif, which interacts with the spike protein of the virus (Aldaais et al., 2021).

The MERS-CoV E protein has a single α-helical transmembrane domain (Surya et al., 2015). This transmembrane domain can form pentameric ion channels in membranes. Similar to the E proteins of SARS-CoV and SARS-CoV-2, the MERS-CoV E protein also has a C-terminal PBM. However, unlike the E proteins from SARS-CoV and SARS-CoV-2, the PBM of MERS-CoV E protein does not interact with the host cell protein PALS1 (Javorsky et al., 2021).

There was a recent study focused on the determination of the structural properties of NL63 (Sučec et al., 2024). The study found that the TMD of the E protein (ETM) of NL63 adopts an α-helical conformation. Interestingly, they found that upon pH decrease or the presence of Ca2+ ions, the ETM of NL63 does not show much change in its water accessibility, whereas the water accessibility of the SARS-CoV-2’s ETM increases upon the same conditions. These functional differences can be attributed to the structural differences between the two viruses. As discussed in the paper, NL63 ETM possesses a 7DDN9 motif, which compared to the corresponding motif (7EET9) present in the SARS-CoV ETM, is less able to respond to the changes in pH and Ca2+ ions due to differences in sidechain charge and lengths. As shown in sequence alignment in **Figure 3**, 7EET9 motif is present in SARS-CoV and SARS-CoV-2, while missing in less pathogenic HCoVs (HKU1, OC43,NL63, 229E). The previous structural studies conducted on the SARS-CoV ETM identified three Phe residues positioned three residues apart from each other in its hydrophobic segment, which played a role in the channel’s gating function. In the case of the NL63 ETM, three Phe residues are positioned successively, and thus may be unable to participate in gating function. In **Figure 3**, it is shown that three Phe residues positioned three residues apart from each other are common to SARS-CoV and SARS-CoV-2, whereas less pathogenic HCoVs do not share this feature. The experimental conditions used with low pH and high Ca2+ concentrations highly resemble the conditions in the ERGIC compartment, in the membrane of which E protein is located. Thus, lower pathogenicity of coronaviruses can partly be linked to their reduced viroporin activity.

From the sequence alignment in **Figure 3**, we can see that M residue located in the NTD and L, C and P residues located in the CTD of the E protein are conserved across all HCoVs. Additionally, three Phe residues positioned three residues apart from each other are common to SARS-CoV and SARS-CoV-2 (F22, F25, F28) whereas less pathogenic HCoVs do not share this feature. This may partly explain the enhanced pathogenicity of SARS-CoV and SARS-CoV-2 in relation to other less virulent HCoVs.

### 3.2 Function

**Figure 5:**
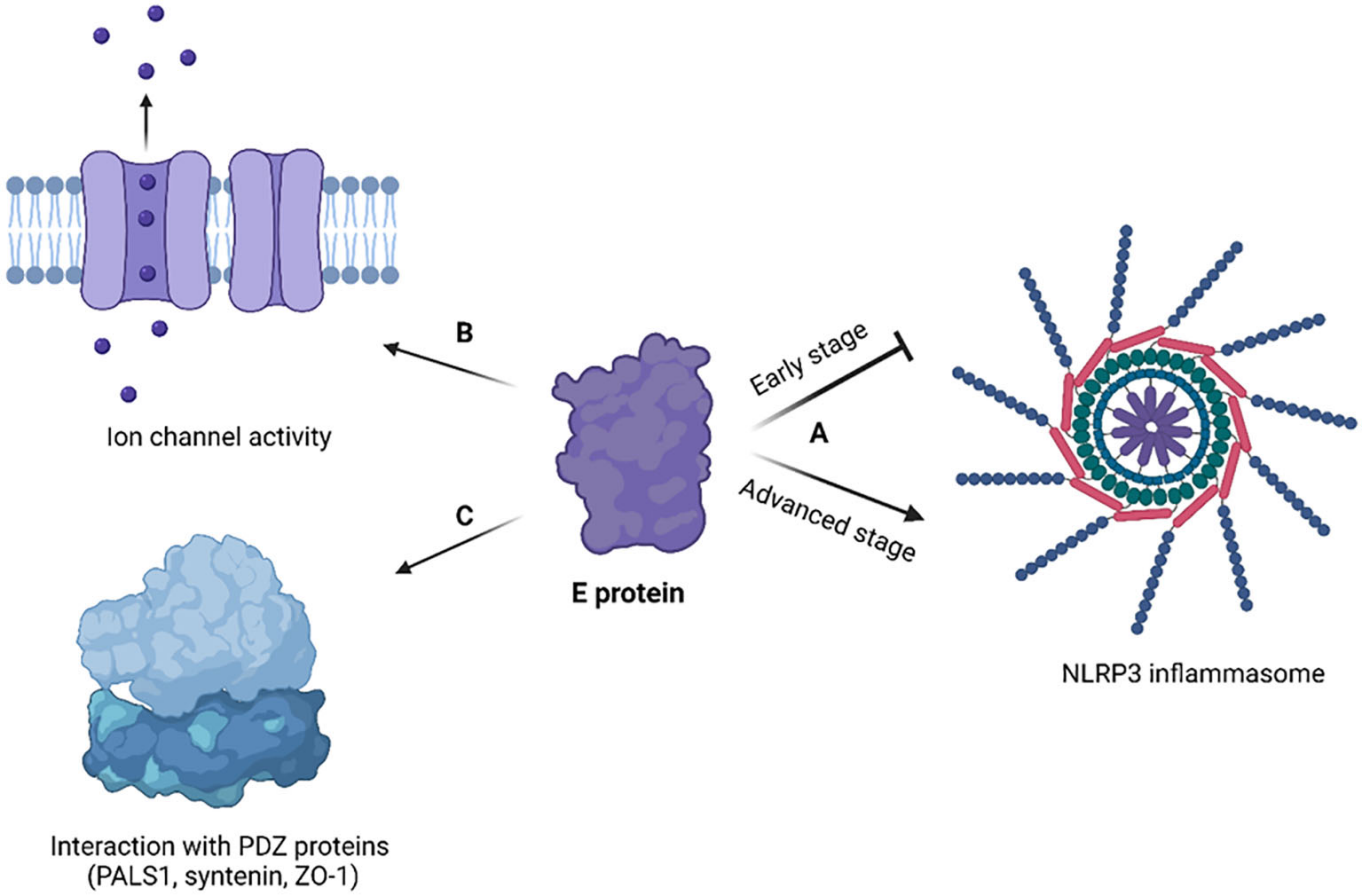
Cellular effects of coronavirus E protein. **(A)** In early stage of disease, E protein dampens the activation of NLRP3 inflammasome, while in advanced stage it activates NLRP3 inflammasome and exacerbates immune response; **(B)** E protein has ion channel activity and enables Ca^2+^ ions transport across ERGIC membrane; **(C)** E protein interacts with human PDZ proteins (PALS1, syntenin, ZO-1) through its PBM. The figure is created using BioRender.

When compared to other coronavirus structural proteins, the E protein is unique in the sense that only a small proportion of it forms virions, while most gets incorporated into the membrane of the ERGIC in infected cells (Venkatagopalan et al., 2015). Although research on the coronavirus E protein is quite scarce, based on the available data, it is certain that the E protein plays crucial roles in the virus’s lifecycle (**Figure 5**; **Table 3**).

**Table 3:** Cellular effects of human coronavirus E protein.

HCoV	Cellular effect	Cells
SARS-CoV-2	Induction of pyroptotic cell death (Chai et al., 2021; Zheng et al., 2021)	THP-1, Vero E6, 16HBE, A549, HeLa, CaCO-2
	Induction of expression of inflammatory cytokines (TNF-α, IL-6) and chemokines (CXCL9 and CCL12) (Xia et al., 2021)	RAW264.7
	Activation of TLR2 signaling (Planès et al., 2022)	HEK cells stably transfected with TLR2
	Interaction with human PALS1 and ZO1 (Javorsky et al., 2021)	*In vitro* assay with recombinant proteins
	Induction of necroptosis and inflammatory response (Baral et al., 2023)	Lung and colon cells
	Activation of TLR 2/4 and downstream JNK signaling leading to increased Cl^-^ concentration and inflammation (Xu et al., 2024)	BEAS-2B cells, human primary cultured airway epithelial cells (hPAECs)
SARS-CoV	Ion channel (IC) activity and activation of NLRP3 inflammasome (Nieto-Torres et al., 2015)	Vero E6
	Interaction with human PALS1 (Teoh et al., 2010)	HEK 293T
	Interaction with Bcl-xL and subsequent T-cell apoptosis (Yang et al., 2005)	Jurkat T-cells
	Interaction with human syntenin and subsequent p38 MAPK activation (Jimenez-Guardeño et al., 2014)	Vero E6
OC43	Infectious virus production (Stodola et al., 2018)	HRT-18

#### 3.2.1 Virus assembly and budding

The viral E protein of SARS-CoV-2 plays a crucial role in retaining the S protein inside infected cells, specifically localizing it to the membranes of the ERGIC (Endoplasmic Reticulum-Golgi Intermediate Compartment) or the Golgi apparatus by slowing down the host cell’s secretory pathway (Boson et al., 2021). Furthermore, the E protein, in collaboration with M protein, facilitates the N-glycosylation of the S protein through a mechanism that operates independently of its intracellular retention (Boson et al., 2021). This coordinated action between the E, M, and S proteins is essential for the proper assembly of virus-like particles.

The E protein, in conjunction with the M protein, facilitates the budding of the virus within the ERGIC. In a study, atomistic molecular dynamics simulations were conducted to understand this process better (Collins et al., 2021). The simulations utilized refined structural models of the SARS-CoV-2 M protein dimer and E protein pentamer. The results showed that while multiple M protein dimers acted together to induce global membrane curvature through protein-lipid interactions, the E protein pentamers helped to keep the membrane planar. This cooperation between the E and M proteins is fundamental for the budding process to occur effectively.

Another study found that the monomeric E protein both generates and senses membrane curvature, preferring to localize with its C-terminus at the convex regions of the membrane (Kuzmin et al., 2022). This characteristic is also observed when the protein is in its pentameric form. The localization to curved regions is deemed favorable for the assembly of E protein oligomers, and the induction of curvature is suggested to facilitate the budding of viral particles.

In summary, additional research is required to ascertain if the SARS-CoV-2 E protein can directly cause membrane curvature. Nonetheless, the role of the E protein in budding is unequivocal, particularly for its CTD.

#### 3.2.2 Host cell effects

Xia et al. showed that SARS-CoV-2 E protein forms a pH-sensitive cation channel causing cell death resembling pyroptosis (Xia et al., 2021). In addition, the E protein was shown to provoke robust immune responses, notably upregulating cytokines (TNF-α, IL-6) and chemokines (CXCL9, CCL12) both *in vitro* and *in vivo*, mirroring the cytokine storm observed in COVID-19 patients (Xia et al., 2021)​.

SARS-CoV-2 E protein transfection triggered necrotic cell death and inflammatory response in both lung and colon cells (Baral et al., 2023). The cellular effects of E protein have been mediated by the activation of the receptor interacting protein kinase 1 (RIPK1), which is a necroptotic marker. Subsequently, RIPK1 promotes the phosphorylation of NF-κB, a key transcription factor involved in inflammation. Recently, there was an intriguing finding that SARS-CoV-2 E protein switches the innate immune system to a tolerant state upon secondary infections (Geanes et al., 2024). Though initially E protein activates the innate immune system via its interaction with TLR-2, its long-term effect is to make monocytes and macrophages unresponsive to pathogens, contributing to immune dysregulation. This could potentially explain why patients with severe forms of COVID-19 are susceptible to secondary infections. SARS-CoV-2 E protein has been shown to activate TLR-2/4 and subsequently JNK signaling, which leads to a high intracellular Cl^-^ concentration through increased expression of phosphodiesterase 4D (PDE4D) (Xu et al., 2024). The increased level of Cl- ions further drive inflammation by enhancing the phosphorylation of serum/glucocorticoid regulated kinase 1 (SGK1) (Xu et al., 2024). Interestingly, blocking SGK1 or PDE4D helps to mitigate the inflammatory response triggered by E protein. This highlights novel therapeutic targets to treat COVID-19 related inflammation.

Previous research on SARS-CoV showed that strains lacking the E protein couldn’t activate the NF-κB pathway, reducing inflammatory cytokine production (DeDiego et al., 2014). Recent studies on SARS-CoV-2 confirmed this finding, emphasizing the significant role of viral E in eliciting robust immune responses both *in vitro* and *in vivo* (Xia et al., 2021).

The SARS-CoV-2 E protein can induce the release of inflammatory cytokines like TNF-α and IFN-γ, and activate the NLRP3 inflammasome (Zheng et al., 2021). This activation is linked to the ion channel property of the E protein which facilitates ion transport, providing an activation signal for the NLRP3 inflammasome assembly. Moreover, the interaction between the SARS-CoV-2 E and Toll-like receptor 2 (TLR2) was identified, which further underscores the role of E protein in innate immune responses. The modulation of the NLRP3 inflammasome by E protein varies across different infection stages (Yalcinkaya et al., 2021). Initially, it may suppress the host NLRP3 inflammasome response to viral RNA but might enhance the NLRP3 inflammasome response in later infection stages.

The E protein disrupts cell polarity by interacting with certain connexins. The interaction primarily occurs at a site known as the PDZ-Binding Motif (PBM) in the E protein, which comprises the last four carboxy-terminal amino acids (DLLV) (Chai et al., 2021). PDZ domains are common protein interaction modules that recognize short amino acid sequences at the C-terminus of target proteins. These domains are found in a variety of connexins, and they can recognize and interact with several human cell junction proteins including PALS1, ZO-1, and syntenin. PALS1 is a crucial protein associated with tight junctions and plays a vital role in maintaining epithelial polarity. Interaction of SARS-CoV E protein with PALS1 has been associated with lung epithelial cell destruction in SARS patients (Teoh et al., 2010). Comparatively, the E protein of SARS-CoV-2 showed an increased affinity for PALS1’s PDZ domain which could be a contributing factor to SARS-CoV-2’s increased virulence (Toto et al., 2020).

A study by Chai et al. used cryo-electron microscopy to visualize the complex structure formed by PALS1 and SARS-CoV-2 E protein, revealing how the DLLV motif of E protein recognizes a hydrophobic pocket formed by PDZ and SH3 domains of PALS1 (Chai et al., 2021). This interaction disrupts the apical cell polarity complex, which could lead to loosened and leaky lung epithelial junctions, promoting local viral spread and immune cell infiltration into lung alveolar spaces.

## 4 Structure and function of M protein

### 4.1 Structure

**Figure 6:**
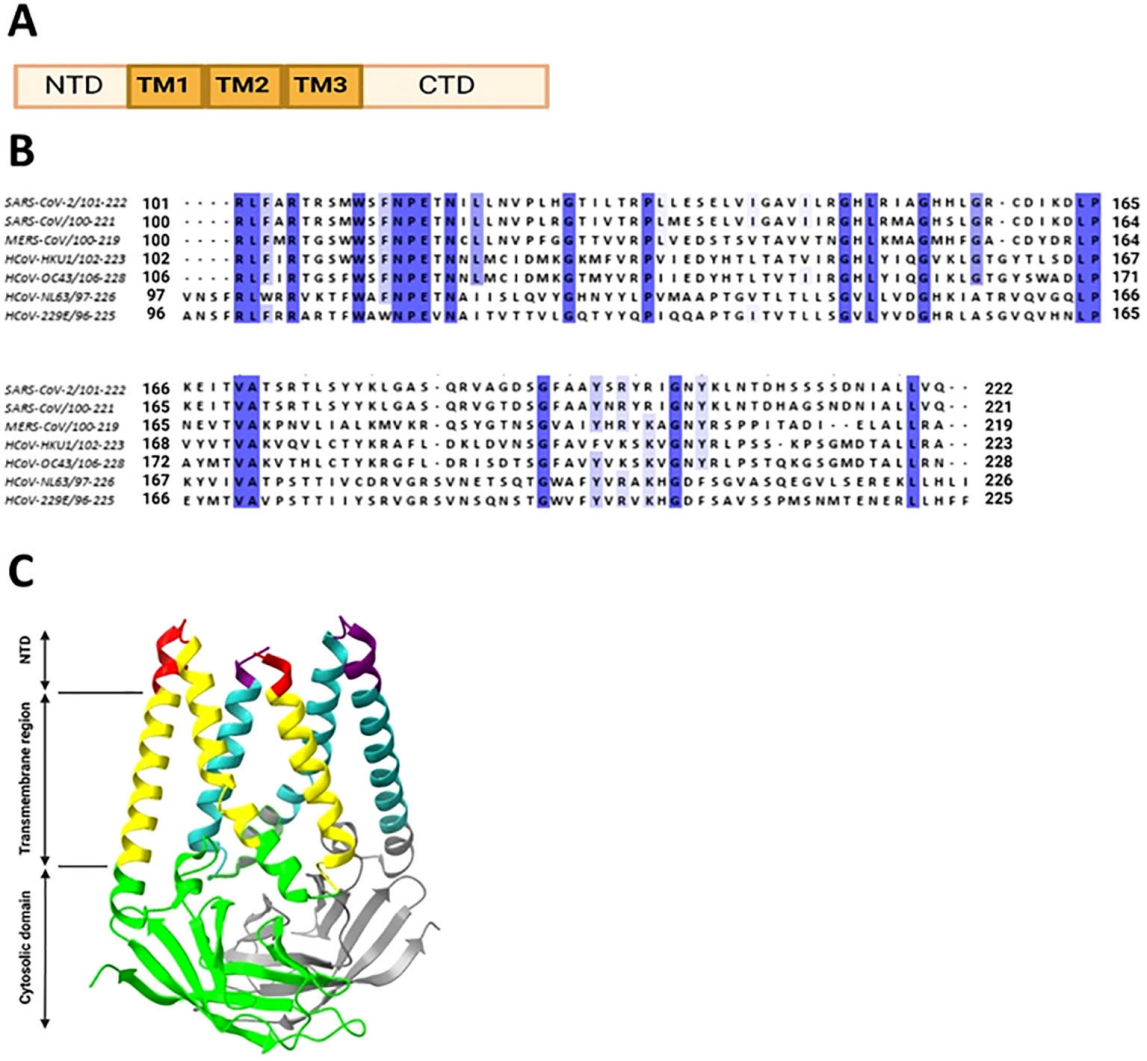
Coronavirus M protein structure. **(A)** Structural organization of coronavirus M protein. **(B)** Multiple sequence alignment of M protein CTDs from human coronaviruses was conducted in Jalview. The invariant residues are highlighted in dark purple, while conserved ones are in light shades. **(C)** Cryo-EM structure of SARS-CoV-2 M protein homodimer. N-terminal domains are color coded in red and purple, transmembrane domains in yellow and cyan, C-terminal domains in green and gray, respectively, for two chains.

The M protein, being the predominant structural protein of the virus, plays a critical role in driving the assembly of the virus and initiating the budding process from the membrane (Z. Zhang et al., 2022). Structurally it consists of a short exterior N-terminal domain (NTD), three transmembrane domains and a long C-terminal domain, located inside the virion, as shown in **Figure 6**.

The M protein of SARS-CoV-2 creates a dimer resembling a mushroom, made up of two three-helix bundles that are transmembrane and domain-swapped, along with a pair of intravirion domains (Zhang et al., 2022). Additionally, the M protein forms into more complex oligomeric structures. The N-terminal ectodomain of M contains the single N-glycosylation site (Voß et al., 2009).

The study found that the MERS-CoV M protein localizes intracellularly within the trans-Golgi network (Perrier et al., 2019). Two motifs in the C-terminal domain were identified as being crucial for this specific localization. The first motif identified is a diacidic DxE ER export signal, essential for the protein’s movement out of the ER. The second motif (199KxGxYR204) is crucial for retaining the M protein within the trans-Golgi network. Mutations in these motifs resulted in altered intracellular localization.

The researchers identified two specific epitopes, Mn2 and Md3, from the M protein, which were found to stimulate strong cellular immunity responses (Liu et al., 2010). The study employed various methods like *in vitro* refolding, T2 cell-binding assays, and analysis in transgenic mice and human cells. The results indicated that the M protein, particularly the identified epitopes, plays a crucial role in cellular immune responses against SARS-CoV. This discovery is significant for vaccine design, as it highlights the M protein as a potential target for eliciting strong and protective immune responses. The study’s findings contribute to a deeper understanding of the immunogenicity of SARS-CoV and offer insights for developing effective vaccines.

The cytoplasmic tail of the M protein is necessary for the localization of the S protein to the Golgi region when both proteins are coexpressed in cells (McBride and Machamer, 2010). A specific tyrosine residue (Y195) within the M protein’s cytoplasmic tail is crucial for the interaction with the S protein. Mutation of Y195 to alanine disrupts the S-M interaction, preventing the retention of the S protein at the Golgi and affecting the S protein’s carbohydrate processing and surface levels. The study concludes that the Y195 residue plays a significant role in the assembly of infectious SARS-CoV by facilitating efficient S-M interaction. This interaction is involved in the assembly process of the viral life cycle. As shown in the **Figure 6B**, Y195 is present in all of the HCoVs, except for the HKU1.

The sequence alignment of C-terminus domain of M protein shows 20 conserved amino acids (highlighted in purple in **Figure 6B**).

### 4.2 Function

The SARS-CoV-2 M protein interacts with the mitochondrial antiviral-signaling protein (MAVS), a central adaptor protein in the innate immune response, to inhibit the antiviral immune response (Fu et al., 2021). This interaction impairs MAVS aggregation and its recruitment of downstream signaling components. The two N-terminal transmembrane domains of the M protein are essential for its inhibition of MAVS-mediated signaling.

The membrane glycoprotein of SARS-CoV-2 initiates caspase-dependent apoptosis (a form of programmed cell death) by binding to PDK1, a protein involved in cell survival signaling, and inhibiting the PDK1-PKB/Akt pathway (Ren et al., 2021). This pathway is crucial for cell survival, and its disruption leads to cell death. The nucleocapsid protein N of SARS-CoV-2 was found to enhance this apoptosis caused by M by facilitating the interaction between M and PDK1, thus further weakening the PDK1-PKB/Akt signaling. Notably, when the interaction between M and N was disrupted by specially designed peptides, the inhibitory effect on the PDK1-PKB/Akt pathway was reversed, preventing the apoptosis enhanced by N. Molecular simulations study found key residues in SARS-CoV-2 M protein C-terminal domain responsible for its interaction with nucleocapsid and spike proteins, which include Phe103, Arg107, Met109, Trp110, Arg131, and Glu135 (Mahtarin et al., 2022).

The SARS-CoV-2 M protein inhibits the production of IFN-I by promoting the degradation of TBK1 via K48-linked ubiquitination, which leads to the enzyme’s breakdown (Sui et al., 2021). This degradation impairs the assembly of a protein complex essential for IFN-I production, resulting in a decreased immune response.

The M protein stabilizes the pro-apoptotic protein BOK (B-cell lymphoma 2 (BCL-2) ovarian killer) by inhibiting its ubiquitination, promoting its mitochondrial translocation, and inducing apoptosis (Yang et al., 2022). Additionally, it was shown that the M protein interacts with the BH2 domain of BOK, amplifying its apoptotic effect. *In vivo* experiments demonstrate that M protein expression via a lentivirus can cause lung cell apoptosis and increase pulmonary permeability in mice, suggesting the M protein’s role in exacerbating lung injury in COVID-19.

Interaction between the SARS-CoV-2 membrane protein and the human Proliferating Cell Nuclear Antigen (PCNA) protein was demonstrated (Zambalde et al., 2022). This interaction is of interest for its potential as a therapeutic target in COVID-19 treatment. PCNA serves as an indicator of DNA damage and plays a crucial role in both the replication and repair of DNA. The study found that the SARS-CoV-2 M protein facilitates the transport of PCNA from the nucleus to the cytoplasm, which may be a viral strategy to manipulate cell functions. This translocation and interaction are proposed as possible targets for COVID-19 therapy.

The recent study found that SARS-CoV-2 M protein interferes with cleavage of S protein by furin, a step important for viral entry to host cells via fusion. There are two mechanisms employed by M protein for this role: one is to interact with S protein and render it in the cytoplasm not allowing it to localize to the membrane; the other one is to bind with furin and thus inhibit its enzymatic activity (Xiang et al., 2024).

SARS-CoV M protein physically interacts with a component of the immune signaling pathway (IKKb) and suppresses the activation of NF-kB (Fang et al., 2007). This suppression leads to a reduction in Cyclooxygenase-2 (Cox-2) expression, a protein involved in inflammation and immune response. The research suggests that the SARS-CoV virus might use this mechanism to evade the host’s immune system, contributing to the pathogenesis of SARS. The M protein of SARS-CoV specifically suppresses the type I interferon (IFN) production, a critical part of the innate immune response to viral infections (Siu et al., 2014). This effect was not observed with the M protein from the human coronavirus HKU1. The first transmembrane domain (TM1) of the SARS-CoV M protein is essential for this suppression. The study showed that this domain alone is sufficient to mediate the suppression of IFN production. TM1 interacts with several key immune signaling molecules like RIG-I, TRAF3, TBK1, and IKKe. Additionally, it was observed that TM1 is necessary for targeting the M protein to the Golgi apparatus in cells, which is a crucial step in the suppression mechanism. These findings provide insight into the molecular mechanisms SARS-CoV uses to evade the host’s immune response, contributing to its pathogenesis.

A study on SARS-CoV M protein was conducted to identify amino acid residues critical for virus-like particle (VLP) production (Tseng et al., 2013). Dileucine motif in the M protein’s endodomain tail is crucial for incorporating the nucleocapsid protein into virus-like particles. Cysteine residue C158 is significant for the M protein’s interaction with the nucleocapsid, essential for virus-like particle formation. Mutations at W19, W57, P58, W91, Y94, F95 reduce virus-like particle yields, indicating these residues’ importance in M protein secretion and assembly.

The MERS-CoV M protein interferes with the host’s innate antiviral defense by inhibiting the activation of the interferon regulatory factor 3 (IRF3), but not NF-κB (Lui et al., 2016). This inhibition occurs through the M protein’s interaction with TRAF3, a key adapter protein, which disrupts the association between TRAF3 and TBK1, leading to decreased IRF3 activation. Additionally, the study reveals that the N-terminal transmembrane domains of the MERS-CoV M protein are essential and sufficient for this inhibitory effect, while the C-terminal domain is largely dispensable. The C-terminal 199KxGxYR204 and 211DxE213 sequences in the membrane protein of the MERS coronavirus play a vital role in the assembly of infectious viruses (Desmarets et al., 2023).

The HCoV-OC43 M protein significantly suppresses the activation of IFN-β promoter, interferon (IFN)-stimulated response element (ISRE), and nuclear factor kappa B response element (NF-κB-RE), thereby reducing the expression of antiviral genes (Beidas and Chehadeh, 2018).

**Figure 7:**
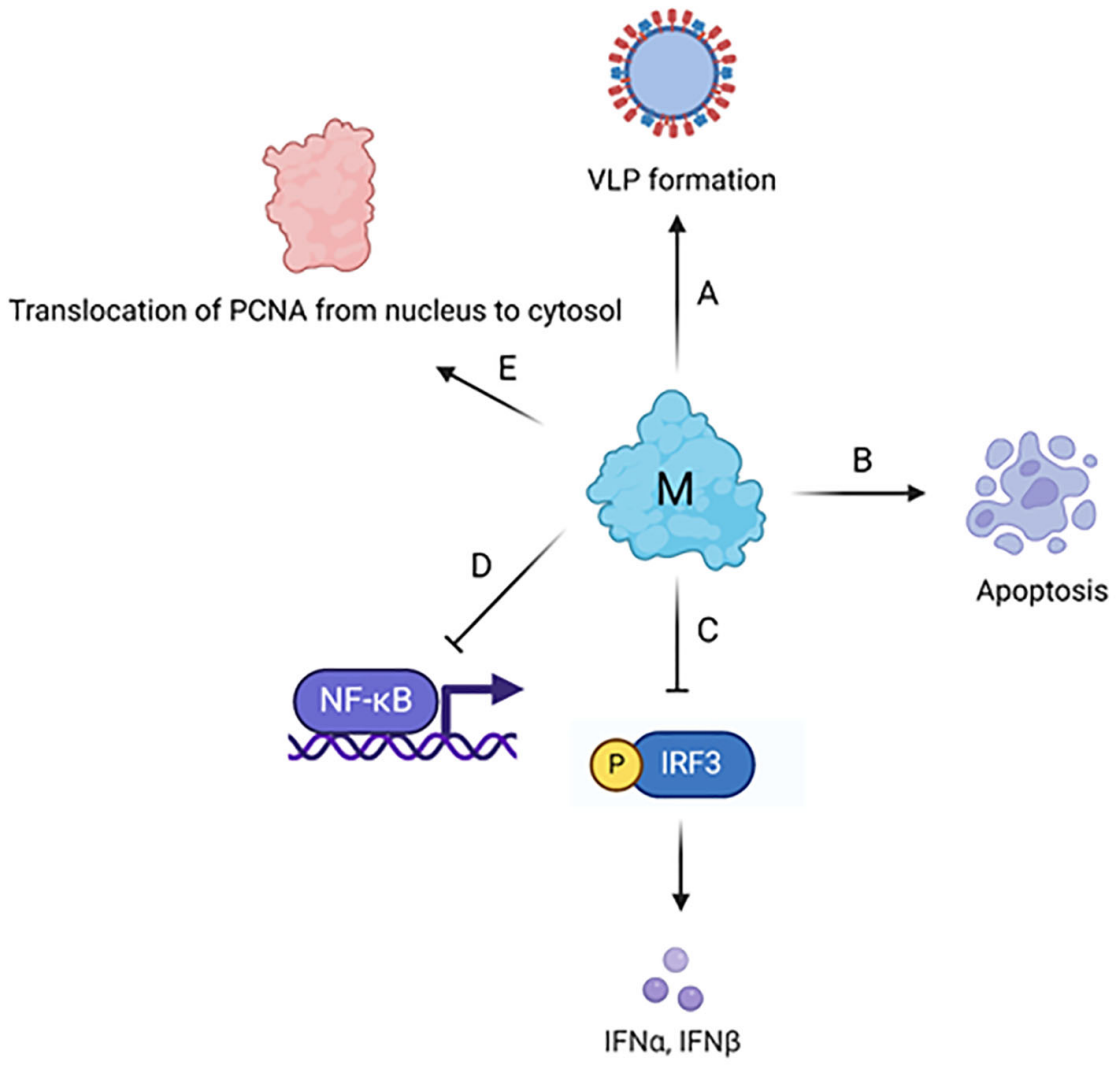
Cellular effects of coronavirus M protein. **(A)** M protein plays a major role in the assembly and formation of virus-like particle via its interaction with other structural proteins and genomic RNA. **(B)** M protein induces mitochondrial apoptosis in lung epithelial cells via the stabilization of B-cell lymphoma 2 (BCL-2) ovarian killer (BOK) and its translocation to mitochondria. **(C)** M protein inhibits TBK1-dependent phosphorylation of IRF3, which further leads to the suppression of type I interferon (IFN) production. **(D)** M protein inhibits NF-κB activation through physically interacting with IκB kinase (IKK). **(E)** The interaction between M protein and human PCNA leads to the translocation of PCNA from nucleus to cytosol. The figure is created using BioRender.

The M protein of HCoV-NL63 facilitates attachment to host cells by interacting with heparan sulfate proteoglycans (HSPGs), a class of cellular receptors (Naskalska et al., 2019). The interaction is mediated by amino acids 153 to 226 located in the C-terminal domain of the M protein. The summary of M protein cellular functions are presented in **Figure 7** and **Table 4**.

**Table 4:** Cellular effects of M protein.

HCoV	Cellular effect	Cells
SARS-CoV-2	Interaction with human Proliferating Cell Nuclear Antigen protein (Zambalde et al., 2022)	Vero E6, HEK293T
	Induction of mitochondrial apoptosis (Yang et al., 2022)	NCL-H292, HEK293T, EA.hy926, Lewis lung cancer
	Interaction with nucleocapsid and spike proteins (Kumar et al., 2023)	protein docking assay
	Suppression of MAVS-mediated innate antiviral response (Fu et al., 2021)	HEK-293
	Induction of caspase-dependent apoptosis due to the interaction with PDK1 (Ren et al., 2021)	Vero E6, HEK393T, HepG2
	TBK1 degradation through K48-linked ubiquitination (Sui et al., 2021)	HEK293T
SARS-CoV	Suppression of type I interferon (Siu et al., 2014) production	HEK293, 293FT
	Suppression of NF-kB activation (Fang et al., 2007)	Vero E6, HeLa
	Interaction with S protein (McBride and Machamer, 2010)	HeLa, HEK293T
	Interaction with N protein and VLP formation (Tseng et al., 2013)	293T, HeLa
	Induction of apoptosis (Chan et al., 2007)	HEK293T
MERS-CoV	Inhibition of the interferon regulatory factor 3 (Lui et al., 2016)	HEK-293
	Formation of infectious viral paricles (Desmarets et al., 2023)	Huh-7
OC43	Reduced transcriptional activity of ISRE, IFN-β promoter, and NF-κB-RE (Beidas and Chehadeh, 2018)	HEK-293
NL63	Interaction with heparan sulfate proteoglycans (Naskalska et al., 2019)	LLC-Mk2

## 5 Structure and function of N protein

### 5.1 Structure

**Figure 8:**
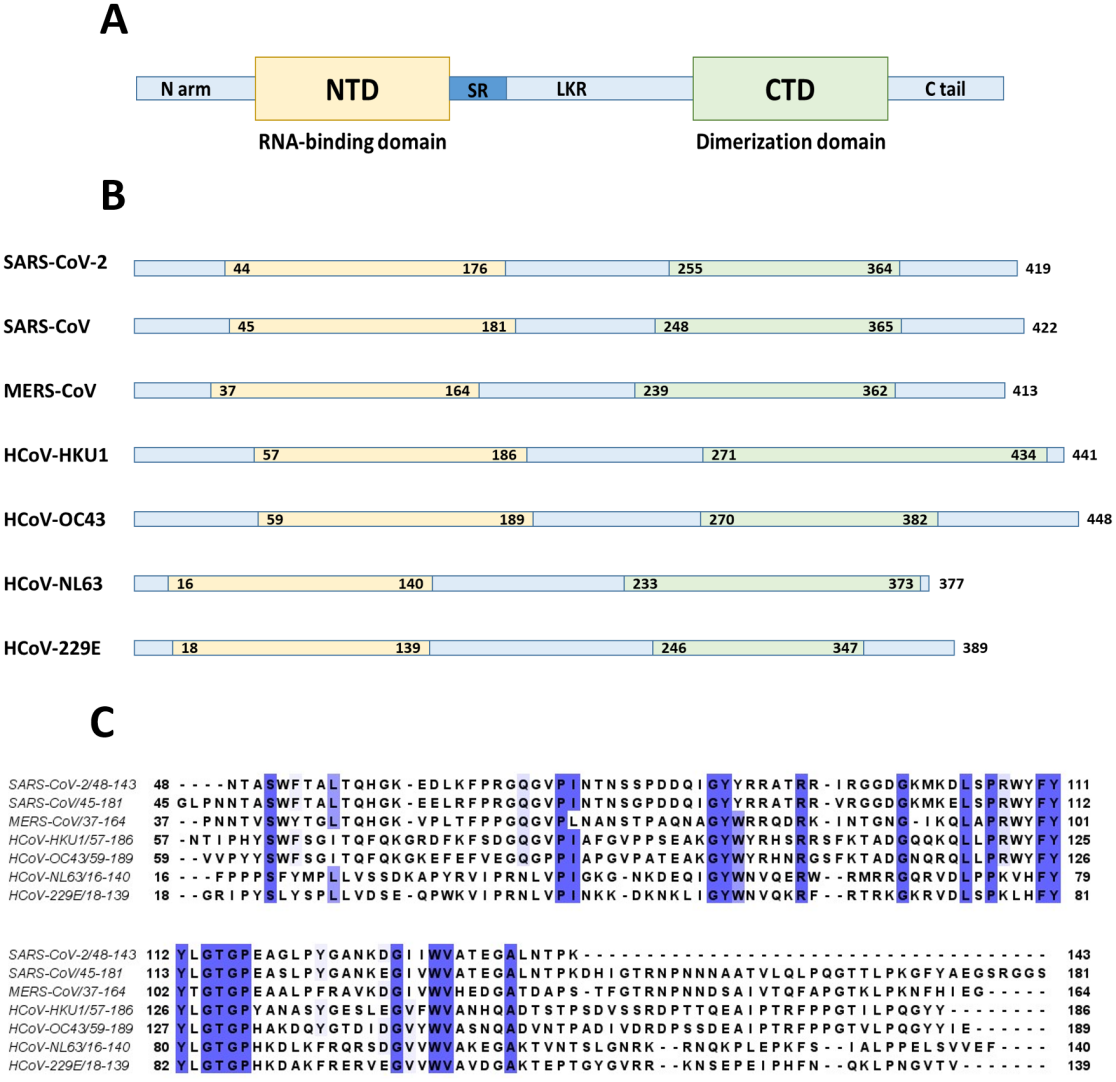
N protein structure. **(A)** NTD and CTD are represented as yellow and green boxes respectively separated by unstructured linker region (LKR) with serine-arginine rich part (SR). **(B)** The entire N protein length as well as lengths of NTD and CTD regions are shown for seven human coronaviruses. **(C)** Clustal sequence alignment of N protein NTDs from human coronaviruses. The invariant residues are highlighted in dark purple, while conserved ones are in light shades. The image is built with a help of Jalview program. The alignment suggests low degree of sequence conservation among different coronaviruses.

Nucleocapsid protein (N protein) has modular organization with two structural components: N-terminal Domain (NTD) and C-terminal Domain (CTD) - separated by intrinsically disordered linker region (LKR) (Wu et al., 2021; Huang et al., 2004; McBride et al., 2014) (**Figure 8**). At the N- and C-termini of the protein there are two intrinsically disordered regions (IDR): N-terminus and C-tail. NTD is referred to as RNA-binding domain, though other domains also have abilities to bind RNA molecules (Kang et al., 2020; Huang et al., 2004). The sequence of NTD as well as its length varies in different coronaviruses (**Figure 8**; **Table 5**). The molecular weight of N protein is within 45 to 60 kDa range. HCoV-OC43 has the longest N protein containing 448 amino acids, whereas HCoV-NL63 has the shortest N protein with 377 amino acids. Despite difference in length, the secondary structures of NTDs of different coronaviruses are well preserved, these include centrally located five-stranded antiparallel β-sheet core domain with RNA-binding site within (Jayaram et al., 2006). RNA-binding region is enriched in basic and aromatic amino acids. Basic residues, such as arginine and lysine, help to neutralize negative charge on phosphate groups of nucleotides. Hydrophobic amino acids interact with nucleotide bases.

**Table 5:** Structural comparison of N protein from different coronaviruses.

Coronavirus	3D structure	PDB	UniProt	Ref.
SARS-CoV-2	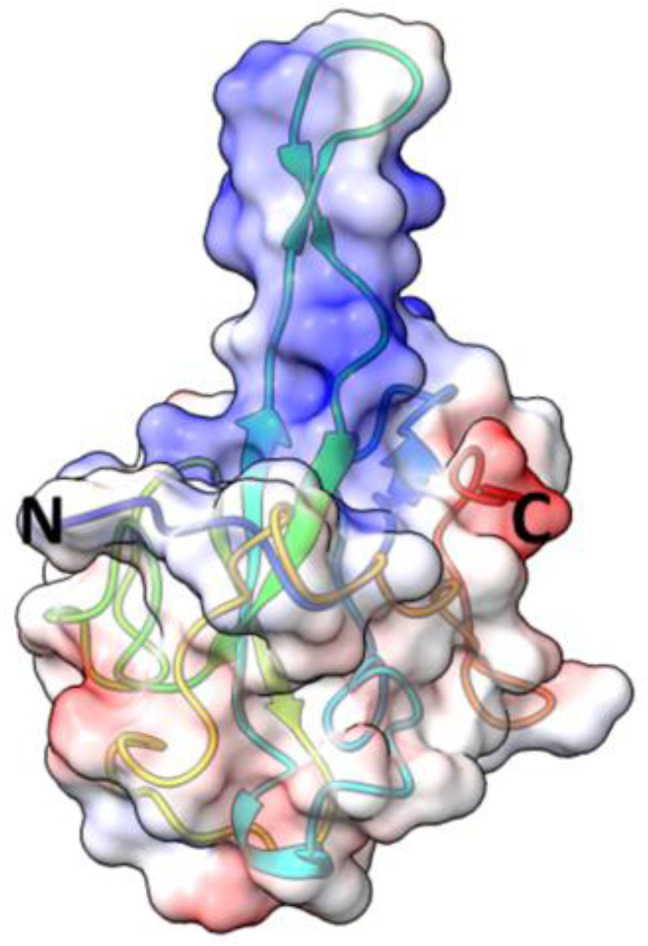	7CDZ	P0DTC9	(Peng et al., 2020)
SARS-CoV	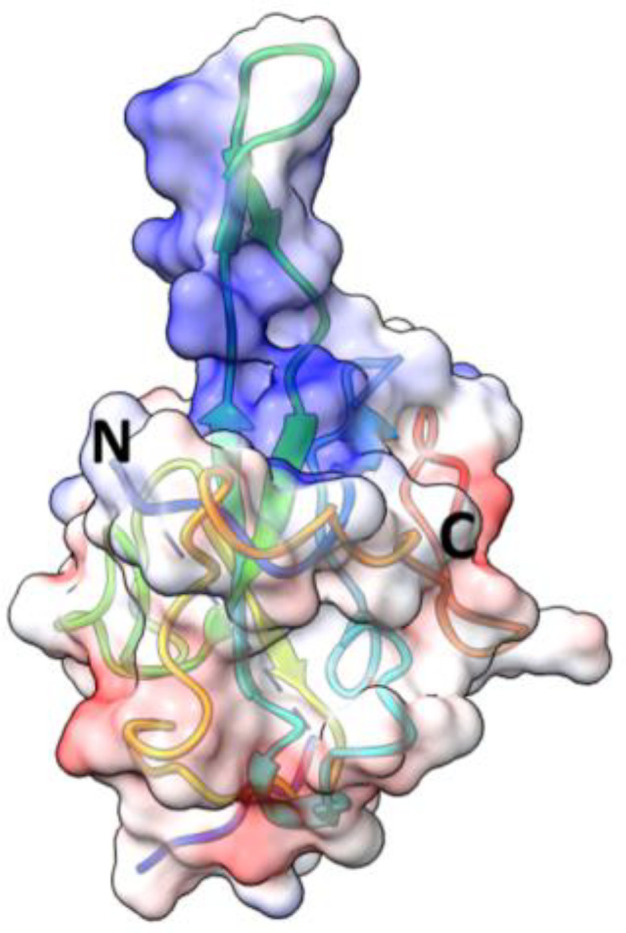	2OFZ	P59595	(Huang et al., 2004)
MERS-CoV	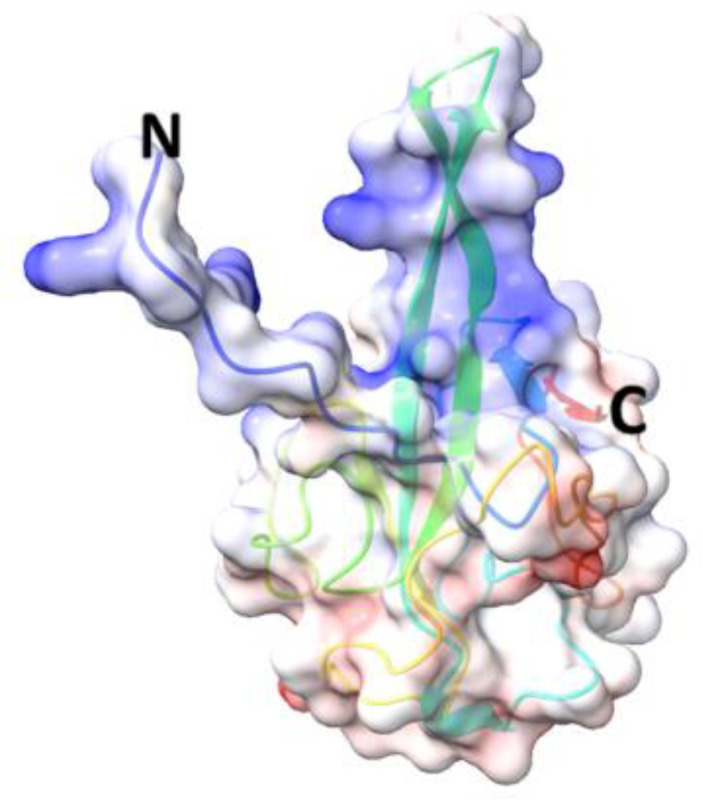	4UD1	K9N4V7	(Papageorgiou et al., 2016)
HCoV- HKU1	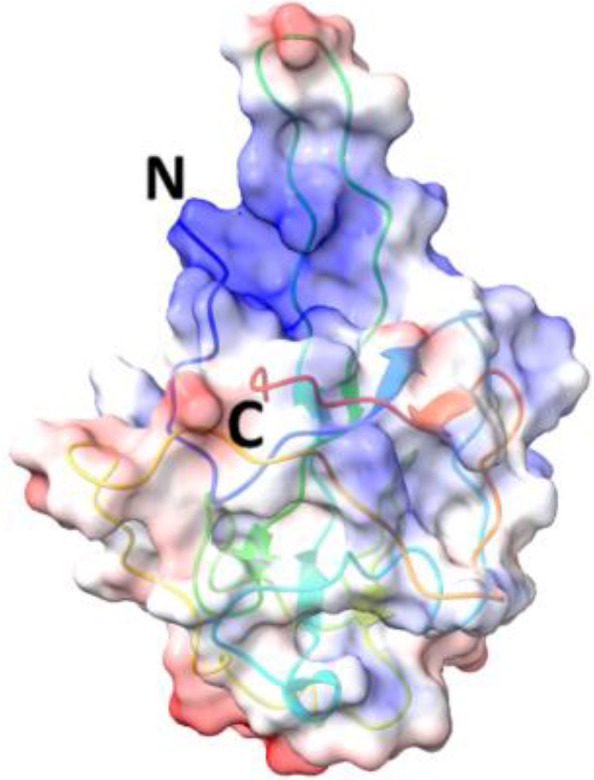	7N45	Q5MQC6	
HCoV-OC43	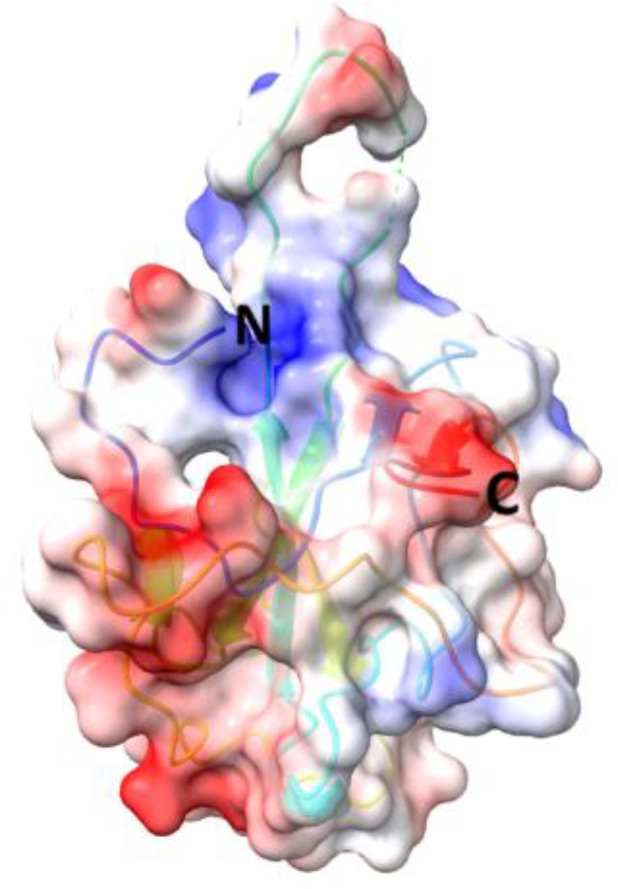	4J3K	P33469	(Chen et al., 2013)
HCoV-NL63	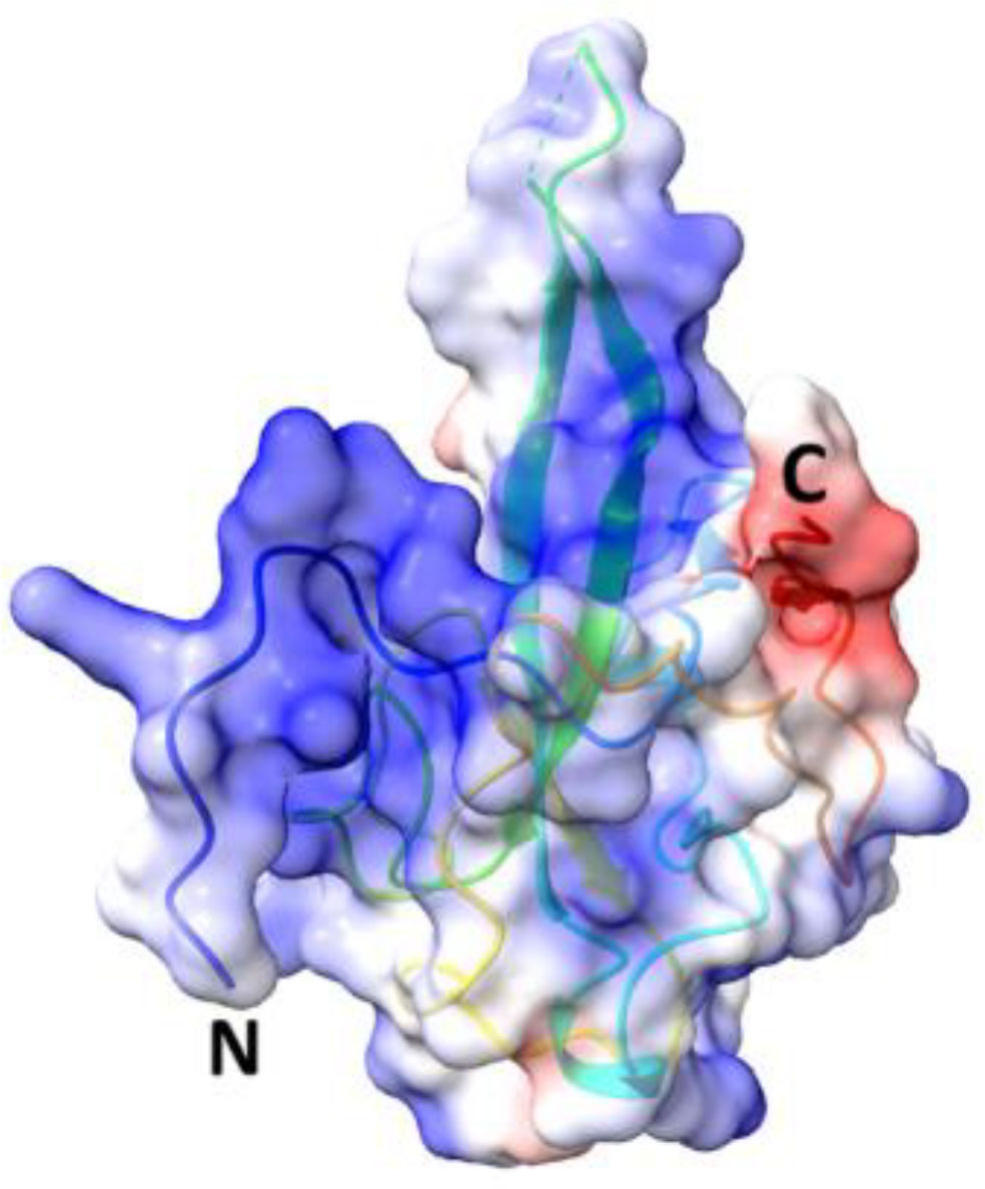	5N4K	Q6Q1R8	(Szelazek et al., 2017)
HCoV-229E	NA	NA	P15130	

LKR region is positioned between NTD and CTD, and it is within an intrinsically disordered region (IDR). It demonstrates flexible structure, and is capable of interacting with nucleic acids and proteins (Wu et al., 2023). At its N-terminal LKR has serine/arginine region, a potential phosphorylation site. The phosphorylation status affects the function of N protein, and in particular, its nuclear translocation through association with host cell 14-3-3 protein (tyrosine 3-monooxygenase/tryptophane 5-monooxygenase activation protein) (Surjit et al., 2005). N protein is a target for multiple kinases, such as cyclin-dependent kinase (CDK), glycogen synthase kinase, mitogen-activated protein kinase (MAPK), and casein kinase II (Wu et al., 2009; Surjit et al., 2005; Tugaeva et al., 2021). SR-region is important for viral replication, removal of SR region in SARS-CoV N protein decreases the number of infectious virus particles within infected cells (Tylor et al., 2009). Despite its disordered structure, linker region is important for interaction between N protein and non-structural protein 3 (nsp-3), which is a part of viral replication-transcription complex (Verheije et al., 2010). This interaction is important for viral infectivity (Hurst et al, 2013). There are several studies demonstrating the role of the linker region in oligomerization of N protein (Peng et al., 2008; Chang et al., 2014; Luo et al., 2005). It was shown, for example, that removal of 184-196 residues in SARS-CoV virus abolishes self-polymerization of N protein (He et al., 2004a).

CTD is hydrophobic in nature, and is rich in α-helices. It plays crucial role in N protein self-assembly into homo-dimers or homo-oligomers (Lo et al., 2013; Chang et al., 2006; Huang et al., 2004). The domain responsible for dimerization is evolutionary preserved among different groups of coronaviruses. The structure of CTD of SARS-CoV-2 is similar to SARS-CoV, MERS-CoV, and HCoV-NL63 CTDs. This similarity is especially well seen in a conservative basic groove, which is believed to contribute to RNA binding.

At the N and C termini of coronaviral N proteins there are two additional IDRs. These are N-arm and C-tail regions (McBride et al., 2014). The analysis of the structure of C-tail suggests that it can form instantaneous helical structure (Zhao et al., 2022), which facilitates N protein oligomerization (Cubuk et al., 2021). C-tail facilitates viral packaging, and it is involved in interaction with M protein (Masters, 2019).

N protein is a polymer that can self-assemble into high-order structure. The first step in self-assembly is a dimerization of CTD. In SARS-CoV-2 residues 247-364 are important for CTD dimerization (Ye et al., 2020). Following dimerization, the protein assembles into homotetrameric structure. In SARS-CoV-2 disordered C-terminal residues 365-419 play important role in this assembly. The last step in viral self-assembly is formation of high-order structure where multiple homotetramers are wrapped by viral RNA.

Nucleocapsid protein of SARS-CoV-2 is 46kDa, and 419 aa long, with approximately 40% being in a disordered position (Perdikari et al., 2020). The structure of NTD was solved (PDB: 6YI3). NTD of SARS-CoV-2 N protein spans 133 aa residues (from 44 to 176 aa residues) (Wu et al., 2023). It has a structure of right-handed fist (Peng et al., 2020) (Bai et al., 2021; Kang et al., 2020; Dinesh et al., 2020), where core structure made up of four antiparallel β strands (β1, β2, β5, and β6) is positioned between short 3_10_ helix and β-hairpin loop made up of β3 and β4 strands. The RNA binding groove is between β-hairpin and β-strand core. The basic residues R92, R107, and R149 were shown to be important for RNA binding (Bai et al., 2021). There is a high degree conservation in RNA-binding site between N proteins of different coronaviruses (Wu et al., 2023).

The structure of CTD of SARS-CoV-2 N protein was solved (PDB: 6YUN, 7CE0, 6ZCO, 7CZ0), and it was shown to have a dimeric organization ( Zhou et al., 2020; Zinzula et al., 2021; Peng et al., 2020; Bai et al., 2021). Each monomer contains five α-helices (α1 to α5), two β-strands, and two 3_10_ helices. Two β strands of each subunit combine to form four antiparallel β-strand structure within a core of a dimer. The dimeric organization is reinforced by hydrogen bonds and hydrophobic interactions. CTD was shown to be important for RNA-induced liquid phase separation (LLPS) which affects function of NF-κB (Wu et al., 2021).

The linker region of SARS-CoV-2 N protein contains a leucine-rich region (210 – 246 residues) important for LLPS (Zhao et al., 2022). SR region of LKR was shown to possess RNA-binding abilities (Wu et al., 2021). Mutagenic studies of R203 demonstrated its role in protein-protein interaction (Syed et al., 2021; Zhao et al., 2022).

Nucleocapsid protein of SARS-CoV is 422 aa long. NTD as well as CTD together with disordered regions are capable of binding RNA (Chang et al., 2009). The structure of N terminus was solved (Saikatendu et al., 2007; Huang et al., 2004). NTD spans 137 aa residues (45 - 181). NTD is rich in lysine and arginine, which are believed to recognize and bind a short 32-nucleotide-long stem –loop structure at the 3’-end of the viral genome (Huang et al., 2004). Among the basic and aromatic residues important for RNA binding, R94 and Y122 were suggested to be critical (McBride et al., 2014). Linker region is structurally disordered, and contains residues (168-208) interacting with viral M protein (He et al., 2004b), as well as region (161-210) interacting with human cellular hnRNP A1 protein (Luo et al., 2005). Crystal structure of CTD of SARS-CoV (270-370 residues) revealed that protein consists of five α-helices, a 3_10_ helix, and two β-strands (Chen et al., 2007; Yu et al., 2006). CTD has dimeric structure in a solution (Takeda et al., 2008; Chang et al., 2006). Dimerization occurs when a β-hairpin of one monomer is brought into the cavity of another monomer, with a formation of an antiparallel β-sheet made up of four β-hairpins (Yu et al., 2006). Within CTD, the loop between W302 and P310 was shown to be important for binding to cyclophilin A (Luo et al., 2004). SARS-CoV C-tail residues (aa 365 – 419) interact with viral M protein by electrostatic interaction (Luo et al., 2006).

Nucleocapsid protein of MERS-CoV is 413 aa long (~40 kDa). The structure of NTD was solved (PDB: 4UD1, 6KL2). Overall, the domain has a globular shape with a central β-sheet core formed from three antiparallel β-strands, and a β-hairpin projecting from the core. The β-hairpin is charged positively and contributes to the RNA binding (Fan et al., 2005).

For nucleocapsid protein of HCoV-OC43 it was shown that Arg106 plays important role for interaction with RNA (Chen et al., 2013). Besides it, S64, G68, Y126, and R164 were shown to be critical for interaction between NTD of HCoV-OC43 N protein and AMP via hydrogen bonds, while Y124 was shown to stabilize the interaction via π-π stacking (Lin et al., 2014). Overall, within HCoV-OC43 N protein there are three RNA-binding regions: residues 1-173, 174-232, and 233-300 (Huang et al., 2009).

Nucleocapsid protein of HCoV-NL63 interacts with nucleic acid with a region that spans 2 -144 amino acids.

Nucleocapsid protein of HCoV-229E was shown to possess binding affinities toward different types of nucleic acids, such as single-stranded DNA, double-stranded DNA, and ssRNA (Tang et al., 2005). The role of the C-terminal tail in N protein oligomerization was shown (Lo et al., 2013).

### 5.2 Function

#### 5.2.1 Viral functions

Nucleocapsid protein is the most abundant protein of the coronaviruses. The main function of this protein is to encompass the viral RNA into ribonucleocapsid (Wu et al., 2023). Besides this structural role, N protein also is important for RNA replication and transcription (Schelle et al., 2005; Egloff et al., 2004). NTD of N protein is capable of melting dsRNA which is important during transcription (McBride et al., 2014; Zúñiga et al., 2010; Grossoehme et al., 2009) and viral replication. Due to its destabilization effect on duplex RNA, N protein was suggested to have RNA chaperon activity (McBride et al., 2014). In addition, N protein could bind to Nsp3 (non-structural protein) to form replication-transcriptional complexes (Khan et al., 2021). Moreover, the dimerization of N protein is important aspect for virus assembly through protein-protein interactions with other structural proteins, and with host membrane envelope. Association of CoV N protein with ER-Golgi apparatus is connected to budding of virus from the cell. Moreover, it has been suggested that N protein is capable of liquid-liquid phase separation, which is a mechanism of concentrating proteins and oligonucleotides promoting viral replication (Savastano et al., 2020). N protein is immunogenic, and evokes strong immune response (Leung et al., 2004). It was shown that N protein plays role in host cell signaling processes.

#### 5.2.2 Cellular effects

Host cells have numerous ways of dealing with RNA-virus infections. Viruses on the other hand develop multiple mechanisms to escape host cell immune response. N protein was shown to contribute to such escape mechanisms. One of the way of escaping host cellular immune reaction of SARS-CoV-2 is to block pyroptosis (Kayagaki et al., 2015). LPS-induced pyroptosis operates via activation of caspase-11 with a subsequent cleavage of Gusdermin D (GSDMD), which leads to NLRP3-dependent activation of caspase-1 and subsequent proteolytic maturation of IL-1β. This cascade results in increased cellular permeability and cellular death (Kayagaki et al., 2015). It was suggested that the N protein of SARS-CoV-2 binds GSDMD and blocks it further in pyroptosis pathway (Ma et al., 2021), inhibiting cell death. At the same time, and contrary to the above, it was shown that N protein can assist M protein in activation of apoptosis via PKB/Akt signaling (Ren et al., 2021).

**Figure 9:**
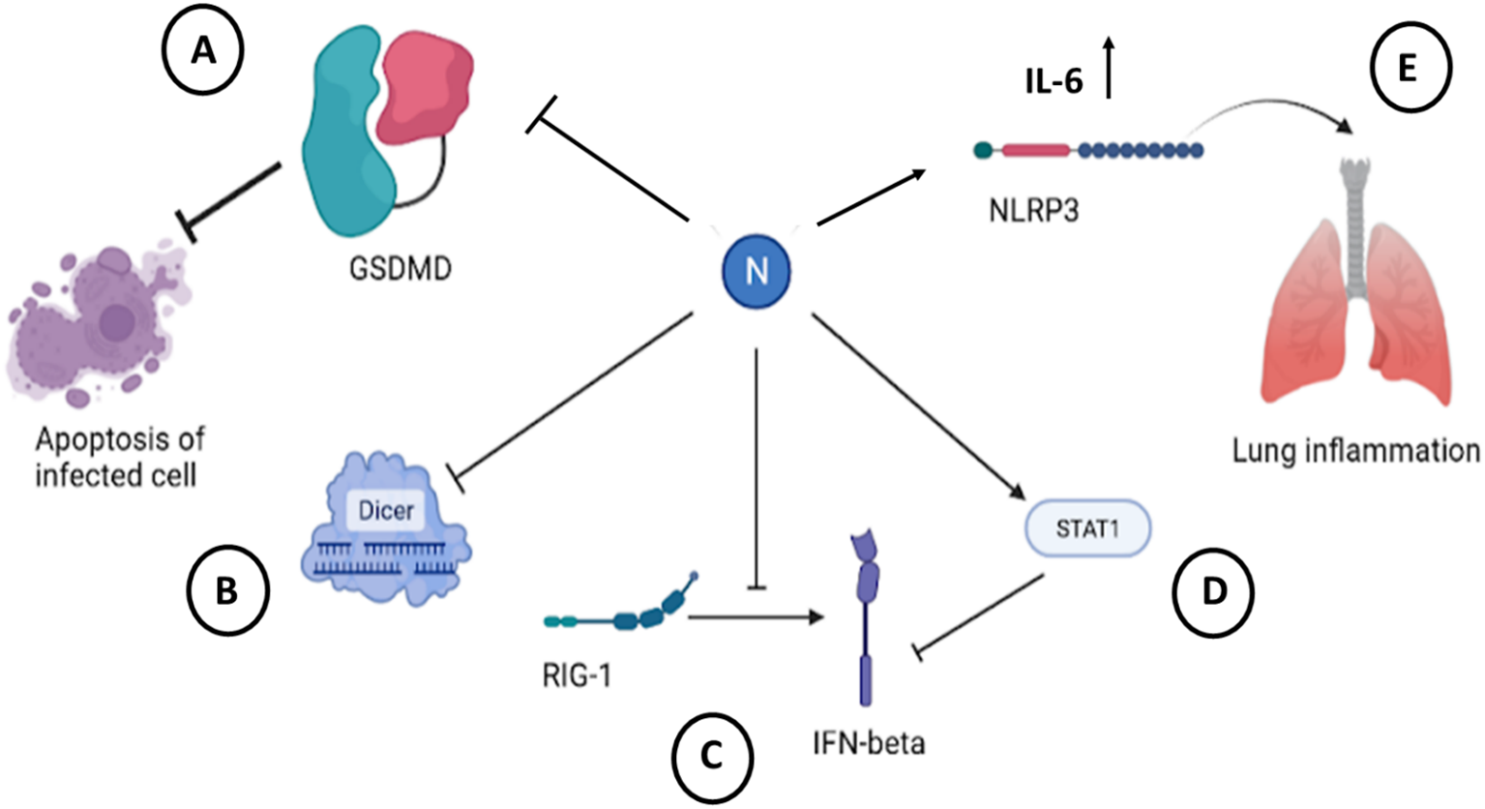
Cellular effects of SARS-CoV-2 N protein. **(A)** Binding and inactivating of GSDMD blocks apoptosis of cells infected with SARS-CoV-2 (Ma et al., 2021). **(B)** Sequestration of dsRNA by N protein precludes recognition and subsequent cleavage by Dicer (Mu et al., 2020a). **(C)** Interaction with RIG-1 pathway leads to a decreased secretion of IFN-β (Oh and Shin, 2021; Chen et al., 2021). **(D)** Binding to STAT1 decreases production of IFN-β in infected cells (Mu et al., 2020b). **(E)** Binding to NLRP3 induces inflammasome activation and initiates inflammation in infected tissue (Pan et al., 2021). The figure is created using BioRender.

Another cellular mechanism to inactivate viral infection is RNA interference. SARS-CoV-2 and SARS-CoV N protein can antagonize cellular host antiviral activities by suppressing the RNAi mechanism (Cui et al., 2015; Xu et al., 2024; Bai et al., 2021) at the level of siRNA synthesis as well at the level of silencing complex formation. N-protein sequester double stranded RNA to prevent cuts from Dicer (**Figure 9**).

One of the main immune mechanisms against viral infection is interferon-mediated pathway. Coronavirus escape this immune defense by inhibiting an interferon system. N-protein of SARS-CoV-2 is identified as an antagonist of interferon. The expression of IFN-β in infected cells was shown to be inhibited (Li et al., 2020). This is due to interaction of N protein with RIG-1 through DExD/H domain (Bai et al., 2021; Chen et al., 2021).

Activation of Janus kinase STAT pathway leads to activation of interferon-mediated pathway. It was shown that N protein of SARS-CoV-2 interferes with this process by inhibiting interaction between STAT1 with JAK1, STAT2 with TYK by binding to STAT1/STAT2 and preventing their phosphorylation in 293T cells (Mu et al., 2020a). This leads to inhibition of type I IFN signaling (**Figure 9**; **Table 6**).

**Table 6:** Summary of cellular effects of N protein of seven human coronaviruses.

Coronavirus	Cellular effect	Cell line
SARS-CoV-2	Induce cytokine release ( Zhang et al., 2007; Karwaciak et al., 2021)	A549, primary macrophages, and monocytes
	Inhibition of IFN- β (Chen et al., 2021)	A549, HeLa, and HEK293T
	Inhibition of type I IFN signaling through suppression of phosphorylation and nuclear localization of STAT1 and STAT2 (Mu et al., 2020a)	HEK293T and HepG2
	Suppresses antiviral stress granule formation trough inhibition of PKR kinase and G3BP1 (Zheng et al., 2021)	HEK293T, Vero E6, and HeLa
	Inhibition of host RNAi mechanism (Mu et al., 2020b)	HEK293T
	Interaction with cholesterol transporter NCP1 (García-Dorival et al., 2021)	HEK293T and Vero E6
	Suppresses pyroptosis (Ma et al., 2021)	THP‐1
	Induces inflammasome formation (Pan et al., 2021)	macrophages, dendritic cells, THP-1, HEK293T
SARS-CoV	Actin reorganization and apoptosis in the absence of growth factor (Surjit et al., 2004)	COS-1
	Through interaction with EF1A translation factor inhibits protein synthesis, cytokinesis, and cell proliferation leading to multinucleated cell formation (Zhou Bing et al., 2008)	HEK293T, HeLa, MCF-7, K562
	Inhibits host RNAi mechanism (Cui et al., 2015)	HEK293T, Neuro-2a, L2
	Inhibits synthesis of IFN-β (Kopecky-Bromberg et al., 2007)	A549, HEK293T
	RNA chaperone activity (Zúñiga et al., 2007)	*in vitro* assay with ASBVd hammerhead ribozyme self-cleavage
	Activation of NF-κB (Liao et al., 2005)	Vero E6
MERS-CoV	Interacts with EF1A translation factor resulting in inhibiting cytokinesis (Zhu et al., 2021)	HEK293T and HeLa
	Suppress type I and III interferons (Chang et al., 2020)	A549
	Up-regulate CXCL10 expression (Aboagye et al., 2018)	HEK293 Ft
	Induction of apoptosis (Hocke et al., 2013)	Human lung tissue primary T cells
HCoV-HKU1	RNA chaperon and capsid assembly (Luna Marques et al., 2021)	*In vitro* assay
HCoV-OC43	Upregulates NF-κB expression (Lai et al., 2014) after 24 h of TNF-α treatment	HEK293T
HCoV-NL63	Following viral infection, localizes within cytoplasm, does not affect cell cycle (Zuwała et al., 2015)	HEK293T and LLC-MK2

Besides inhibiting host cell antiviral responses, coronavirus N protein was shown to contribute to inflammatory response. Thus, SARS-CoV-2 N protein promotes release of inflammatory cytokines by activating NLRP3 inflammasome in infected macrophages and dendritic cells (Pan et al., 2021). There are generally four types of inflammasomes: NLRP1, NLRP3, NLRC4, and AIM2 (Broz and Dixit, 2016). NLRP3 is important for functions against viral RNA infections (Wang et al., 2014). The viral nucleocapsid was detected with an ACE receptor, and in cortical tissues with NLRP3 inflammasome (Cama et al., 2021).

Virus-induced inflammation results from a massive cytokine release from infected cells, and N protein seems to play role in it. For example, it was shown that N protein of SARS-CoV-2 upregulates IL-6 expression in monocytes and macrophages at protein and at mRNA level (Karwaciak et al., 2021). Similar to SARS-CoV-2, SARS-CoV virus induces IL-6 upregulation in lung A549 cells following transfection with N-protein coding plasmid (Zhang et al., 2007). This upregulation was mediated by NF-κB transcription factor, where N protein binds NF-κB and transports it to the nucleus to upregulate cytokine transcription. The summary of the described cellular effects of SARS-CoV-2 virus is represented on a **Figure 9** and **Table 6**.

In addition to the above, nucleocapsid of SARS-CoV-2 condensates in host cells (U2OS) with stress granule protein G3BP1, thus suppressing G3BP1 stress granule driven immune response (Lu et al., 2021). It was shown that N protein could be methylated in host cells in R95 and R177 within RGG/RG motifs by methyltransferases which help to suppress stress granules (Cai et al., 2021).

Coronaviruses can interfere with cell cycle through disruption of cell division to prolong interphase stage. This leads to optimal conditions for viral replication. As it was demonstrated for N protein of SARS-CoV, it regulates cyclin-CDK activity. N-protein can be phosphorylated by CDK, and thus, it is a substrate for cyclin-CDK complex (Surjit et al., 2006; Surjit et al., 2005). Besides, N protein exhibits inhibitory effects on S phase kinases, such as CDK4 and CDK6. N protein is illustrated as a competitive inhibitor of CDK4 and CDK6 (McBride et al., 2014). The net result of the SARS-CoV N protein effect on cell cycle is an inhibition of S phase progression.

In addition, N protein of SARS-CoV has been linked to processes which downregulate the host’s translation by binding to elongation factor 1α (eEF1A) (McBride et al., 2014). Elongation factor eEF1A plays multiple roles in various cellular processes, such as translation (Merrick, 1992), protein degradation (Chuang et al., 2005), actin filaments assembly (Gross and Kinzy, 2005), and formation of contractile ring during cytokinesis. Through its interaction with eEF1A, N protein leads to inhibition of protein translation and cytokinesis by blocking F-actin bundling (Zhou Bing et al., 2008). As a result, cell proliferation is slowed. It has been observed that N protein substantially inhibits proliferation of peripheral blood lymphocytes (Zhou Bing et al., 2008).

Also, it has been indicated that N protein can activate AP-1 (activation protein-1) pathway by increasing transcription factors c-Fos, ATF2, CREB-1, and FosB (McBride et al., 2014; He et al., 2003). In addition, SARS-CoV N protein may induce apoptosis in COS-1 monkey kidney cells (Surjit et al., 2004) by downregulating ERK pathway and upregulating JNK and p38 MAPK pathway.

N protein of MERS-CoV inhibits production of IFN type 1 and 3 by inhibiting RIG-1 signaling (Chang et al., 2020). The nucleocapsid protein competes with RIG-1 to interact with TRIM25 ubiquitin. Moreover, the MERS-CoV N protein was shown to interact with host antiviral defenses, up-regulating CXCL 10 gene pathway (Aboagye et al., 2018). The CXCL10 pathway is connected to inflammation, immune dysfunction and tumor development.

HCoV-OC43 virus N protein can affect the host immune system through NF-kB pathway (Lai et al., 2014). N protein was shown to upregulate NF-kB transcription factor by binding and inhibiting miR-9, a negative regulator of NF-kB. Increased level of translation of NFKB1 protein could lead to inflammation.

Surprisingly, HCoV-NL63 N protein was not present in the nucleus of the infected cell, assuming that no alterations to cellular cycle and cellular protein expression was observed (Zuwała et al., 2015). This is in striking contrast with other coronaviruses.

N protein of HCoV-229E was shown to induce release of IFN-β and IP-10 cytokines in embryonal lung fibroblast HFL cells at 24 and 30 hours post infection, and this upregulation is higher compare to the one triggered by MERS-CoV (Lau et al., 2013).

## 6 Conclusions

S protein is a crucial for cell entry. High mutation rate within S protein leads to emergence of new viral strains with improved infectivity and transmissibility, as seen in Alpha, Beta, Gamma, Delta, and Omicron variants of SARS-CoV-2 virus. Composed of two parts, S1 and S2, spike protein binds to a specific receptor on a host cell to initiate viral entry. S1 part contains RBD which has high affinity toward target receptor. S1 part harbors low similarity between different viruses, enabling viruses to recognize different receptor. Thus, SARS-CoV-2, SARS-CoV and NL63 viruses bind ACE2 upon entry. MERS-CoV recognizes dipeptidyl-peptidase 4 as its target. HKU1 and OC43 viruses enter the cells following binding to 9-O-acetylsialic acid, 229E virus gains cellular access through binding to aminopeptidase N. RBD of all viruses but OC43 is found within CTD of S1 subunit. With 1362 amino acids OC43 S protein is the longest in all seven human coronaviruses, while HCoV-229E is characterized by the shortest S protein of 1173 amino acids. Unlike S1 subunit, S2 subunit is highly conserved in different coronaviruses. It contains fusion peptide, necessary for initiation of fusion between viral envelope and host cell membrane. Following cellular entry, S protein contributes to viral pathogenicity. Particularly, spike protein induces ER stress, and leads to the activation of unfolded protein response. It also activate inflammasomes in microglial cells, and upregulates cytokines (Wang et al., 2007; Xue and Feng, 2021; Albornoz et al., 2023).

E protein is a short single-pass transmembrane protein, capable of assembly into homopentameric structure with a pore formation (Mandala et al., 2020), which inside the infected cell could lead to cell death. Through its PDZ-binding motif, E protein is capable of interacting with host cell proteins, such as PALS1 (Chai et al., 2021), syntenin, ZO1, TJP1, PARD3, MLLT4, LNX2 (Zhu et al., 2022; Ávila-Flores et al., 2023). Following such interaction PDZ domains gets sequestered to the Golgi compartment, disturbing cellular physiology. Through interaction with PALS1, E protein disrupts epithelial cells polarity and tight junctions. E protein of SARS-CoV-2 compared to other human coronaviruses was shown to have higher affinity toward PALS1 PDZ domain, which could explain increased virulence of the virus (Toto et al., 2020). E protein was shown to induce immune response from the host cell (Xia et al., 2021). Most of the E protein gets incorporated into the ERGIC membrane within infected cells, where together with M protein, E protein coordinates budding of the virus.

M protein with its three transmembrane domains is highly abundant within viral envelope. It assembles into oligomeric structures, and plays crucial role in virus assembly and budding from the host cell. Via its interaction with S protein, M protein facilitates retention of the S protein within Golgi apparatus (McBride and Machamer, 2010). Following viral entry M protein was shown to inhibit antiviral immune response. The mechanisms involved in such host immune response suppression are virus-specific. SARS-CoV-2 M protein does it through interaction and subsequent inhibition of mitochondrial antiviral-signaling protein (Fu et al., 2021) as well as through inhibition of IFN-1 production (Sui et al., 2021). SARS-CoV M protein reduces immune response by suppression of NF-kB (Fang et al., 2007) activation, and IFN production (Siu et al., 2014). MERS-CoV M protein inhibits IRF3 but not NF-kB (Lui et al., 2016). HCoV-OC43 M protein reduces expression of antiviral genes (Beidas and Chehadeh, 2018).

There is a lack of research on the E and M proteins of less virulent hCoVs, when compared to the two proteins of more virulent HCoVs. However, from literature we have reviewed on E protein it is clear that there are two factors conferring SARS-CoV-1, SARS-CoV-2, MERS-CoV enhanced virulence, which are flexible PBM (which enables to establish stronger protein-protein interaction with host cells) and enhanced ion channel activity. When it comes to the M protein, the number of studies are very few. But we can hypothesize that one factor could be the ability of more virulent HCoVs to suppress type I IFN response and propagate in the host effectively, whereas less virulent HCoVs are deprived of this ability, and as a result they are better controlled by the host.

N protein being the most abundant protein of the coronaviruses, plays crucial role in assembly of the viral genome, as well as in viral replication. Following cellular entry it helps virus to escape antiviral response.

The current review summarizes and highlights the main structural characteristics and functional differences of structural proteins from seven pathogenic to humans coronaviruses. The main findings on structure and function are summarized in form of tables and figures, which could provide insights when novel mechanisms of viral actions are studied or new treatments are thought.
