# Supplementary material for: Structural proteins of human coronaviruses: what makes them different?
Source: Front Cell Infect Microbiol. 2024 Dec 6;14:1458383. doi: 10.3389/fcimb.2024.1458383 (PMC11659265; doi:10.3389/fcimb.2024.1458383)
Supplement: Supplementary file 1 [file Table1.docx]

| Coronavirus | S protein length | NTD | CTD | RBM | S1/S2 cleavage site | S2 cleavage site | Fusion peptide 1 | Fusion peptide 2 | Heptad repeat 1 | Heptad repeat 2 | TM | Ref |
| --- | --- | --- | --- | --- | --- | --- | --- | --- | --- | --- | --- | --- |
| SARS-CoV-2 | 1273 | 14-303 | 334-527 | 437-508 | 680-685 | 808 -820 | 816-837 | 835 -855 | 920 -970 | 1163-1202 | 1214-1237 | NTD, CTD, RBM, FP1, FP2, HR1,HR2, TM domains - UniProt - P0DTC2;  S1/S2 and S2 cleavage sites - (Winger and Caspari 2021) |
| SARS-CoV | 1255 | 14-290 | 321-513 | 424-494 | 662-672 | 797-798 | 798-819 | 817-837 | 902-952 | 1145-1184 | 1194-1227 | NTD, CTD, RBM, FP1, FP2, HR1, HR2, TM domains - UniProt – P59594;  S1/S2 and S2 cleavage sites -  (Yuan et al. 2017);  TM domain – (Broer et al. 2006) |
| MERS-CoV | 1353 | 18-351 | 381-587 | 424-454 | 751-752 | 887-888 | 888-909 | 907-929 | 994-1044 | 1246-1285 | 1295-1326 | NTD, CTD, FP1, FP2, HR1,HR2 domains - UniProt – K9N5Q8;  RBM - (Hatmal et al. 2020) ;  S1/S2 and S2 cleavage sites -  (Yuan et al. 2017);  TM domain - (Zhou et al. 2019) |
| HCoV-HKU1 | 1356 | 14-294 | 325-607 | 500-530 | 757 | 900 | 905-926 | 924-944 | 1005-1055 | 1249-1289 |  | NTD, CTD, FP1, FP2, HR1,HR2 domains - UniProt – Q5MQD0;  RBM - (Saunders et al. 2023);  S1/S2 and S2 cleavage sites -  (Kirchdoerfer et al. 2016) |
| HCoV-OC43 | 1362 | 15-302 | 333-607 |  | 754-758 | 903 | 904-925 | 923-943 | 1004-1054 | 1248-1286 |  | NTD, CTD, FP1, FP2, HR1,HR2 domains – UniProt - P36334;  S1/S2 cleavage site - (Wang et al. 2022);  S2 cleavage site - (Millet and Whittaker 2015) |
| HCoV-NL63 | 1356 | 179-481 | 481-616 | RBM1 493-513 RBM2 531-541 RBM3 585-590 |  | 870 | 934-954 |  | 948-1067 | 1212-1308 |  | NTD, CTD, RBMs - (Wu et al. 2009);  S2 cleavage site - (Millet and Whittaker 2015);  FP1, HR1, HR2 – UniProt – Q6Q1S2 |
| HCoV-229E | 1173 | 17-268 | 297-434 |  |  | 685-686 | 753-773 |  | 767-886 | 1031-1127 |  | NTD, CTD, S2 cleavage site - (Song et al. 2021);  FP1, HR1, HR2 domains – UniProt – P15423 |

**Supplementary Table 1. The lengths and boundaries of S protein structural domains.**

Broer, Rene, Bertrand Boson, Willy Spaan, François-Loïc Cosset, and Jeroen Corver. 2006. “Important Role for the Transmembrane Domain of Severe Acute Respiratory Syndrome Coronavirus Spike Protein during Entry.” *Journal of Virology* 80 (3): 1302–10. https://doi.org/10.1128/JVI.80.3.1302-1310.2006.

Hatmal, Ma’mon M., Walhan Alshaer, Mohammad A. I. Al-Hatamleh, Malik Hatmal, Othman Smadi, Mutasem O. Taha, Ayman J. Oweida, Jennifer C. Boer, Rohimah Mohamud, and Magdalena Plebanski. 2020. “Comprehensive Structural and Molecular Comparison of Spike Proteins of SARS-CoV-2, SARS-CoV and MERS-CoV, and Their Interactions with ACE2.” *Cells* 9 (12). https://doi.org/10.3390/cells9122638.

Kirchdoerfer, Robert N., Christopher A. Cottrell, Nianshuang Wang, Jesper Pallesen, Hadi M. Yassine, Hannah L. Turner, Kizzmekia S. Corbett, Barney S. Graham, Jason S. McLellan, and Andrew B. Ward. 2016. “Pre-Fusion Structure of a Human Coronavirus Spike Protein.” *Nature* 531 (7592): 118–21. https://doi.org/10.1038/nature17200.

Millet, Jean Kaoru, and Gary R. Whittaker. 2015. “Host Cell Proteases: Critical Determinants of Coronavirus Tropism and Pathogenesis.” *Virus Research* 202 (April):120–34. https://doi.org/10.1016/j.virusres.2014.11.021.

Saunders, Nell, Ignacio Fernandez, Cyril Planchais, Vincent Michel, Maaran Michael Rajah, Eduard Baquero Salazar, Jeanne Postal, et al. 2023. “TMPRSS2 Is a Functional Receptor for Human Coronavirus HKU1.” *Nature* 624 (7990): 207–14. https://doi.org/10.1038/s41586-023-06761-7.

Song, Xiyong, Yuejun Shi, Wei Ding, Tongxin Niu, Limeng Sun, Yubei Tan, Yong Chen, et al. 2021. “Cryo-EM Analysis of the HCoV-229E Spike Glycoprotein Reveals Dynamic Prefusion Conformational Changes.” *Nature Communications* 12 (1): 141. https://doi.org/10.1038/s41467-020-20401-y.

Wang, Chunyan, Emma L. Hesketh, Tatiana M. Shamorkina, Wentao Li, Peter J. Franken, Dubravka Drabek, Rien van Haperen, et al. 2022. “Antigenic Structure of the Human Coronavirus OC43 Spike Reveals Exposed and Occluded Neutralizing Epitopes.” *Nature Communications* 13 (1): 2921. https://doi.org/10.1038/s41467-022-30658-0.

Winger, Anna, and Thomas Caspari. 2021. “The Spike of Concern—The Novel Variants of SARS-CoV-2.” *Viruses* 13 (6). https://doi.org/10.3390/v13061002.

Wu, Kailang, Weikai Li, Guiqing Peng, and Fang Li. 2009. “Crystal Structure of NL63 Respiratory Coronavirus Receptor-Binding Domain Complexed with Its Human Receptor.” *Proceedings of the National Academy of Sciences of the United States of America* 106 (47): 19970–74. https://doi.org/10.1073/pnas.0908837106.

Yuan, Yuan, Duanfang Cao, Yanfang Zhang, Jun Ma, Jianxun Qi, Qihui Wang, Guangwen Lu, et al. 2017. “Cryo-EM Structures of MERS-CoV and SARS-CoV Spike Glycoproteins Reveal the Dynamic Receptor Binding Domains.” *Nature Communications* 8 (1): 15092. https://doi.org/10.1038/ncomms15092.

Zhou, Yusen, Yang Yang, Jingwei Huang, Shibo Jiang, and Lanying Du. 2019. “Advances in MERS-CoV Vaccines and Therapeutics Based on the Receptor-Binding Domain.” *Viruses* 11 (1). https://doi.org/10.3390/v11010060.
